# Spectrum of cefepime-taniborbactam coverage against 190 β-lactamases defined in engineered isogenic *Escherichia coli* strains

**DOI:** 10.1128/aac.01699-24

**Published:** 2025-04-01

**Authors:** Tsuyoshi Uehara, Cassandra L. Chatwin, Brittany Miller, Mitchell Edwards, Annie Stevenson, Jenna Colombo, David A. Six, Denis M. Daigle, Greg Moeck, Steven A. Boyd, Daniel C. Pevear

**Affiliations:** 1 Venatorx Pharmaceuticals Inc, Malvern, Pennsylvania, USA; University of Fribourg, Fribourg, Switzerland

**Keywords:** cefepime-taniborbactam, β-lactamases, inhibition spectrum, ceftazidime-avibactam, aztreonam-avibactam

## Abstract

Cefepime-taniborbactam is a β-lactam/β-lactamase inhibitor combination in clinical development for the treatment of Enterobacterales and *Pseudomonas* infections, including carbapenem-resistant Enterobacterales and multidrug-resistant *Pseudomonas aeruginosa*. Taniborbactam is a novel cyclic boronate with direct inhibitory activity against clinically relevant Ambler class A, B, C, and D β-lactamases. To further characterize the spectrum of β-lactamase coverage by cefepime-taniborbactam, we constructed 190 isogenic strains of *Escherichia coli* that constitutively expressed a different β-lactamase. Synthetic codon-optimized genes encoding the mature periplasmic protein linked to the TEM-1 signal sequence were used for optimized expression and periplasmic localization of the β-lactamase. The repertoire of β-lactamases consisted of 50 Ambler class A, 34 class B (metallo), 48 class C, and 58 class D enzymes known to mediate β-lactam resistance in the clinical isolates of Enterobacterales and *P. aeruginosa*. Overall, in the 190 isogenic strains, the MIC_50_/MIC_90_ values were 8/128 µg/mL for cefepime and >128/>128 µg/mL for ceftazidime. Cefepime-taniborbactam (MIC_50_/MIC_90_ of 0.25/8 µg/mL) showed greater activity than ceftazidime-avibactam (MIC_50_/MIC_90_ of 4/>128 µg/mL) and similar activity to aztreonam-avibactam (MIC_50_/MIC_90_ of 0.5/4 µg/mL). Cefepime-taniborbactam inhibited strains overproducing metallo-β-lactamases, including clinically important NDM and VIM enzymes, whereas ceftazidime-avibactam showed no coverage. Among the 129 β-lactamase-overproducing strains with increased cefepime MIC ≥16-fold relative to the control strain, taniborbactam potentiated cefepime MIC by ≥8-fold for 113 strains overexpressing β-lactamases (42 Ambler class A, 24 B, 23 C, and 24 D). Cefepime-taniborbactam demonstrated broader activity relative to ceftazidime-avibactam and comparable activity with aztreonam-avibactam in the overall coverage of both serine- and metallo-β-lactamases from all four Ambler classes.

## INTRODUCTION

Cefepime-taniborbactam is a novel β-lactam/β-lactamase inhibitor (BL-BLI) combination currently in clinical development for the treatment of complicated urinary tract infections associated with multidrug-resistant bacterial pathogens (1, 2). In the phase 3 clinical trial, cefepime-taniborbactam was statistically superior to meropenem for the primary composite endpoint at the test of cure visit (3–5). The microbiological activity, pharmacokinetics, efficacy, safety, and tolerability of cefepime-taniborbactam were summarized in a recent review (6).

Cefepime is a fourth-generation cephalosporin that inhibits the growth of susceptible bacteria by covalently binding to penicillin-binding proteins (PBP) required for the synthesis of the bacterial cell wall (7). Taniborbactam (formerly VNRX-5133) is a cyclic boronate β-lactamase inhibitor that restores the antibacterial activity of cefepime against multidrug-resistant (MDR) Enterobacterales and *Pseudomonas aeruginosa* producing β-lactamases. Taniborbactam inhibits both serine- and metallo-β-lactamases, including extended-spectrum β-lactamases and carbapenemases, thereby restoring the activity of cefepime against MDR Gram-negative bacteria (1, 2). In the GEARS global surveillance program, cefepime-taniborbactam inhibited 99.5% of 20,725 Enterobacterales and 96.5% of 7,919 *P*. *aeruginosa* clinical isolates (8). Most isolates resistant to cefepime-taniborbactam possess multiple potential mechanisms of resistance, including carriage of IMP β-lactamases, or alterations in PBP3 (the major target of cefepime), porin deletions (decreased permeability) and efflux upregulation (8, 9). In the presence of chromosomal β-lactam-resistant mechanisms, such as alterations in PBP3 or decreased permeability, NDM β-lactamases reduce susceptibility to cefepime-taniborbactam (10, 11).

Isogenic strain panels expressing diverse β-lactamases are simple yet powerful tools to evaluate the spectrum of susceptibility of a β-lactam to β-lactamases and the spectrum of inhibition of a β-lactamase inhibitor paired with a β-lactam (2, 12–22). The use of an isogenic strain library normalizes the highly variable effects of permeability and efflux on the β-lactam and β-lactamase inhibitor as well as any convoluting effects of multiple β-lactamases (18). In general, β-lactamases from isogenic strain panels are constitutively expressed from a standard multicopy plasmid with a standard promoter, but often, these libraries use native β-lactamase signal sequences and codon usage (23). This can bias the level of heterologous expression and β-lactamase activity in the background strain of the isogenic library. The advent of inexpensive and reliable DNA synthesis enabled the standardization of the β-lactamase signal sequence and codon optimization of each β-lactamase gene (2), further normalizing the expression of diverse β-lactamases in the isogenic library. In this study, we constructed 190 isogenic strains of *Escherichia coli* overproducing a wide variety of β-lactamases present in Enterobacterales and *P. aeruginosa*. Using these strains, we evaluated the effect of individually overproduced β-lactamases on the activity of cefepime-taniborbactam relative to ceftazidime-avibactam as well as each cephalosporin alone to establish the spectrum of inhibitory activity for each combination. In addition, aztreonam, aztreonam-avibactam, piperacillin, and meropenem were tested against these isogenic strains overproducing individual β-lactamases.

## RESULTS AND DISCUSSION

### Design elements in the construction of *E. coli* isogenic strains with optimal expression of β-lactamases in the periplasm

To assess the breadth of inhibitory activity of taniborbactam against β-lactamases, isogenic *E. coli* K-12 DH10B strains were engineered to overproduce each β-lactamase individually. To optimize the expression levels of β-lactamases, plasmids had an origin of replication with a high copy number (pMB1 origin of replication) and carried DNA sequences of the *bla*_TEM-1_ promoter, the signal sequence of TEM-1, and the codon-optimized sequence encoding each mature β-lactamase. For β-lactamases that have been shown or predicted to be lipoproteins (i.e., NDMs), periplasmic active portions were used for plasmid construction. Despite using the identical promoter and signal sequence, it does not exclude the possibility that the levels of produced β-lactamases may vary due to differences in stability of mRNAs or proteins, by using the heterologous signal peptide, or by changing the subcellular localization (e.g., NDMs). Production of each β-lactamase was confirmed by decreased susceptibility to control β-lactam antibiotics known to be substrates of each specific β-lactamase.

To assess the increase in MIC attributable to the expressed β-lactamase, the *E. coli* DH10B strains carrying pTU501 or pTU672 expressing only the TEM-1 signal sequence from the *bla*_TEM-1_ promoter (Fig. S1) were used as controls to determine the baseline MIC in the absence of expression of an active β-lactamase for each antibiotic. Cefepime, cefepime-taniborbactam, ceftazidime, and ceftazidime-avibactam had modal MICs ranging between 0.12 and 0.5 µg/mL in both control strains. The lack of >2-fold MIC decrease for either cefepime or ceftazidime in the presence of β-lactamase inhibitor confirmed the negligible activity of chromosomal AmpC β-lactamase (ESC-1, narrow-spectrum cephalosporinase) at baseline levels. In addition, taniborbactam and avibactam have no significant standalone antibacterial activity under conditions of the broth microdilution assay (2, 24). Thus, the activity of cefepime-taniborbactam and ceftazidime-avibactam combinations relative to that of the cephalosporin partner alone should provide a quantitative measure of potentiation directly reflecting the levels of inhibitory activity of the β-lactamase inhibitor.

### Cefepime-taniborbactam activity against 190 β-lactamases found in Enterobacterales and *P. aeruginosa*

**Fig 1:**
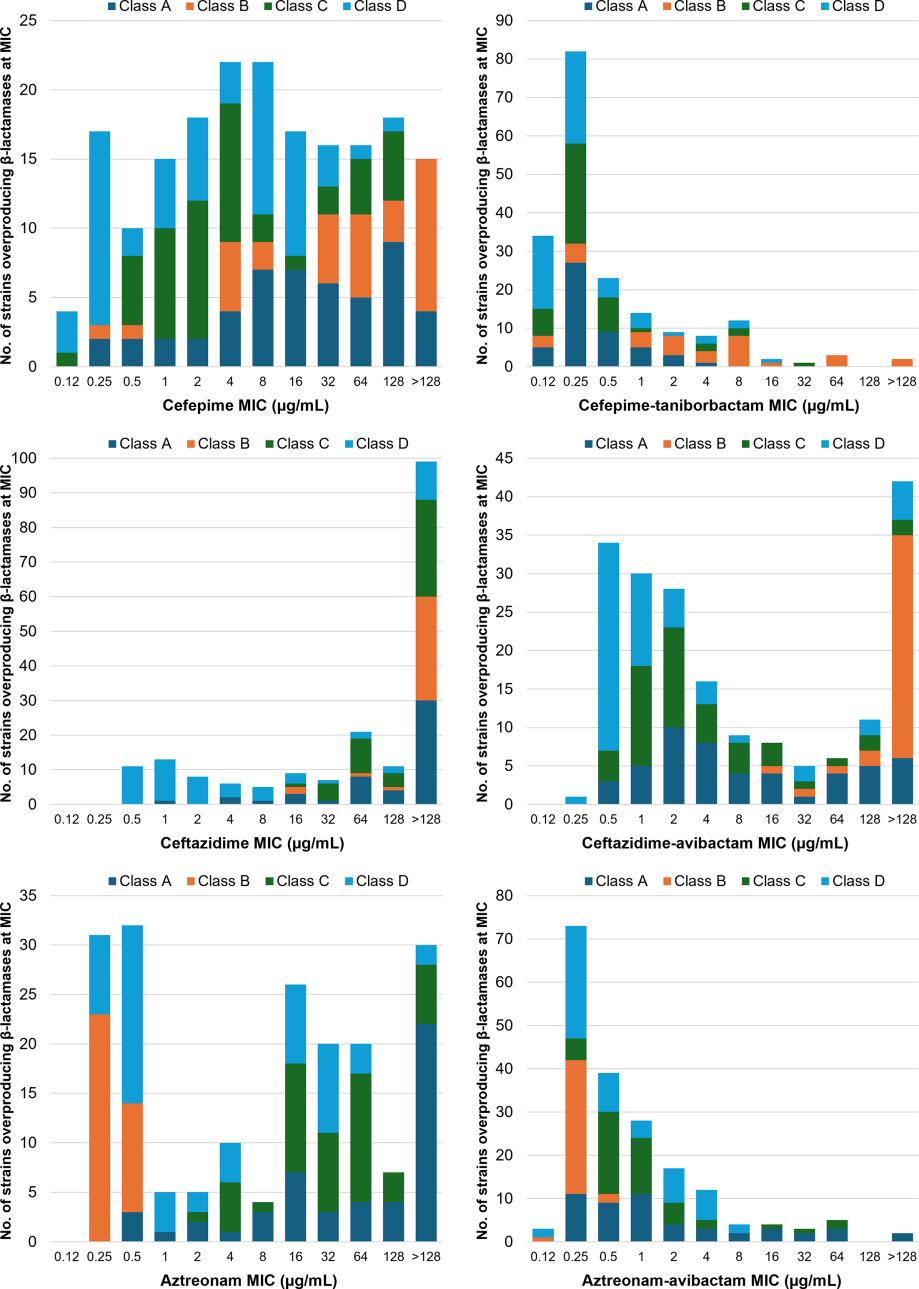
MIC distributions against 190 *E. coli* isogenic strains overproducing β-lactamases, by Ambler class.

To examine the inhibitory activity of taniborbactam, MICs for cefepime and cefepime-taniborbactam were determined against 190 *E. coli* strains overproducing individual β-lactamases, as shown in Table 1. Similarly, ceftazidime and ceftazidime-avibactam MICs were determined against the same set of strains. The evaluated β-lactamases consisted of 50 Ambler class A, 34 class B (metallo), 48 class C, and 58 class D enzymes known to mediate β-lactam resistance in clinical isolates of Enterobacterales and *P. aeruginosa*. Among them, 77 β-lactamases function as a carbapenemase (26 Ambler class A, 34 class B, and 17 class D) according to the Beta-Lactamase DataBase (25). Because the selection of these 190 β-lactamases was largely based on consideration of the prevalence of β-lactamases as well as resistance to β-lactams and BL-BLI in Enterobacterales and *P. aeruginosa*, the list of these enzymes does not reflect the distribution of β-lactamases in clinical isolates. Fig. 1; Tables 2 and 3 show the MIC distributions of cefepime, cefepime-taniborbactam, ceftazidime, ceftazidime-avibactam, aztreonam, and aztreonam-avibactam. Overproduction of all β-lactamases increased the MIC of cefepime, ceftazidime, aztreonam, and/or piperacillin ≥4-fold, confirming the activity of each enzyme in the overproducing strain (Table 1). The fold increase in MIC due to the production of individual β-lactamases was calculated by comparing the MIC against the engineered overproducing strain with that against the respective vector control strain and is shown for each β-lactamase-overproducing strain in Table S1. Because enzymes were tested in an antibiotic-susceptible *E. coli* K-12 strain, the fold increase in MIC reflects only the activity of overproduced enzymes to inactivate β-lactams in the presence or absence of a BLI without other resistant mechanisms present in clinical strains of Enterobacterales and *P. aeruginosa*.

**TABLE 1:** Spectrum of antibacterial activity of cefepime-taniborbactam defined in engineered strains of *E. coli* producing individual Ambler class A, B, C, and D β-lactamases^*a*^^*,^b^*^

*E. coli* DH10B producing	Variant description^*e*^	Ambler class	MIC (µg/mL)								FEP MIC fold decrease by TAN	CAZ MIC fold decrease by AVI	ATM MIC fold decrease by AVI
			FEP	FEP-TAN	CAZ	CAZ-AVI	ATM	ATM-AVI	PIP	MEM			
Signal peptide (pTU501 vector)			0.25	0.12	0.5	0.5	0.25	0.25	2	0.03	2	1	1
Signal peptide (pTU672 vector)^*c*^			0.12	0.12	0.5	0.5	0.25	0.25	2	0.03	1	1	1
TEM-10	TEM-1 R164S E240K	A	**8**	1	**>128**	2	**64**	1	**128**	0.06	8	>64	64
TEM-12	TEM-1 R164S	A	1	0.25	4	1	0.5	0.25	**32**	0.03	4	4	2
TEM-24	TEM-1 Q39K E104K R164S	A	2	0.5	**>128**	**8**	**16**	1	8	0.06	4	>16	16
TEM-72	TEM-1 Q39K M182T G238S E240K	A	**32**	0.25	**>128**	2	**128**	1	**>128**	0.25	128	>64	128
CTX-M-2	CTX-M-2 class	A	**128**	0.25	**32**	2	**128**	0.5	**>128**	0.12	512	16	256
CTX-M-14^*c*^	CTX-M-14/KLUY-1	A	**16**	0.12	4	0.5	**32**	0.25	**>128**	0.03	128	8	128
CTX-M-15	CTX-M-3 class	A	**128**	0.25	**>128**	1	**>128**	0.5	**>128**	0.12	512	>128	>256
CTX-M-219^*c*^	CTX-M-14 P167S T263I	A	**16**	0.5	**128**	2	**16**	0.25	**>128**	0.06	32	64	64
GES-2^*c*^^*d*^		A	**8**	0.25	**128**	4	**16**	0.25	**128**	0.12	32	32	64
GES-4^*c*^^*d*^	GES-2 M62T E104K N170S	A	0.5	0.12	**64**	2	**4**	0.5	**128**	**0.5**	4	32	8
GES-5^*d*^	GES-2 N170S	A	2	0.25	**64**	4	2	0.25	**>128**	**4**	8	16	8
GES-6^*c*^^*d*^	GES-2 E104K N170S	A	**4**	0.25	**>128**	16	**16**	0.5	**>128**	**4**	16	>8	32
GES-11^*c*^	GES-2 N170G G243A	A	**64**	0.25	**>128**	8	**>128**	1	**>128**	**0.5**	256	>16	>128
GES-13^*c*^	GES-2 E104K	A	**16**	0.25	**>128**	16	**>128**	1	**>128**	0.25	64	>8	>128
GES-14^*c*^^*d*^	GES-2 N170S G243A	A	**8**	0.25	**>128**	4	**8**	0.25	**>128**	**2**	32	>32	32
SHV-5		A	**>128**	0.25	**>128**	4	**>128**	2	**>128**	0.12	>512	>32	>64
SHV-12	SHV-5 L35Q	A	**128**	0.25	**>128**	4	**>128**	2	**>128**	0.12	512	>32	>64
VEB-9		A	**128**	0.25	**>128**	**16**	**>128**	**4**	**64**	0.03	512	>8	>32
VEB-14^*c*^	VEB-9 del_T215	A	**>128**	**4**	**>128**	**>128**	**128**	**32**	**128**	0.06	>32	NA	4
VEB-25^*c*^	VEB-9 K234R	A	**>128**	0.5	**>128**	**>128**	**>128**	**>128**	**>128**	0.12	>256	NA	NA
KPC-2^†^		A	**64**	0.25	**64**	1	**>128**	0.5	**>128**	**16**	256	64	>256
KPC-21^*c*^^*d*^	KPC-2 W104R	A	**4**	0.25	**64**	1	**>128**	**16**	**32**	**1**	16	64	>8
KPC-2 L168Q^*c*^^*d*^	KPC-2 L168Q	A	**8**	0.5	**128**	4	**16**	1	**64**	**0.5**	16	32	16
KPC-2 del_172–175^*c*^^*d*^	KPC-2 del_172–175	A	0.25	0.25	**16**	8	0.5	0.25	4	0.03	1	2	2
KPC-14^*c*^^*d*^	KPC-2 del_241–242	A	**32**	**2**	**>128**	**128**	**128**	**4**	**128**	0.12	16	>1	32
KPC-87^*c*^^*d*^	KPC-2 G241A del_T242	A	**16**	**2**	**128**	**64**	**64**	**64**	**32**	**4**	8	2	1
KPC-3^*d*^	KPC-2 H274Y	A	**128**	0.25	**>128**	4	**>128**	0.5	**>128**	**16**	512	>32	>256
KPC-3 A177E^*d*^	KPC-3 A177E	A	**128**	0.25	**>128**	4	**>128**	0.5	**>128**	**16**	512	>32	>256
KPC-31^*d*^	KPC-3 D179Y	A	**32**	1	**>128**	**>128**	**16**	1	**64**	0.25	32	NA	16
KPC-8^*d*^	KPC-3 V240G	A	**128**	0.5	**>128**	**64**	**>128**	2	**>128**	**8**	256	>2	>64
KPC-3 T243A^*d*^	KPC-3 T243A	A	**64**	0.25	**>128**	**8**	**>128**	0.5	**>128**	**8**	256	>16	>256
KPC-3 A177E D179Y^*d*^	KPC-3 A177E D179Y	A	**16**	0.5	**>128**	**>128**	8	2	**64**	0.25	32	NA	4
KPC-32^*d*^	KPC-3 D179Y T243M	A	**16**	0.5	**>128**	**128**	8	0.5	**64**	0.25	32	>1	16
KPC-66^*d*^	KPC-3 del_166–167	A	1	0.12	**64**	2	1	0.25	16	0.03	8	32	4
KPC-109^*c*^^*d*^	KPC-3 Ins_269_KYN	A	**32**	1	**>128**	**128**	**64**	1	**>128**	**0.5**	32	>1	64
KPC-134^*c*^^*d*^	KPC-2 D179Y ins_269NRAPN	A	**32**	1	**>128**	**>128**	2	1	**32**	0.25	32	NA	2
KPC-163^*c*^^*d*^	KPC-2 ins_269KHS	A	**32**	1	**>128**	**128**	**64**	1	**128**	0.25	32	>1	64
PER-1		A	**64**	0.25	**>128**	**16**	**>128**	**16**	**128**	0.12	256	>8	>8
PER-2	PER-1 +33 variants	A	**>128**	0.5	**>128**	**64**	**>128**	**64**	**>128**	0.12	>256	>2	>2
PER-4^*c*^	PER-1 S130T	A	**64**	**2**	**>128**	**>128**	**>128**	**>128**	16	0.03	32	NA	NA
PER-6^*c*^	PER-1 +32 variants	A	**128**	0.5	**>128**	**64**	**>128**	**64**	**>128**	0.12	256	>2	>2
PER-7^*c*^	PER-1 E116Q I241V K242R T285S	A	**128**	0.25	**>128**	**32**	**>128**	**32**	**>128**	0.12	512	>4	>4
PER-14^*c*^	PER-2 S130T	A	**8**	0.25	**>128**	**128**	**16**	**16**	4	0.016	32	>1	1
BEL-1^*c*^		A	0.5	0.12	**64**	0.5	**32**	0.25	**>128**	0.03	4	128	128
BEL-2^*c*^	BEL-1 L162F	A	**4**	0.12	**64**	2	**32**	0.25	**32**	0.03	32	32	128
IMI-1^*c*^^*d*^		A	**4**	0.25	**16**	2	**>128**	**4**	**>128**	**32**	16	8	>32
NmcA^*c*^^*d*^		A	**8**	0.25	**8**	2	**>128**	**8**	**>128**	**64**	32	4	>16
SFC-1^*c*^^*d*^		A	**16**	0.25	**16**	1	**>128**	1	**>128**	**32**	64	16	>128
SME-2^*c*^^*d*^		A	**8**	0.25	**64**	2	**>128**	**8**	**>128**	**32**	32	32	>16
LAP-2		A	0.25	0.25	1	0.5	0.5	0.25	16	0.03	1	2	2
NDM-1^*d*^		B	**128**	**2**	**>128**	**>128**	0.5	0.25	**>128**	**32**	64	NA	2
NDM-3^*d*^	NDM-1 D89N	B	**>128**	**8**	**>128**	**>128**	0.25	0.25	**>128**	**>128**	>16	NA	1
NDM-5^*d*^	NDM-1 V92L M155L	B	**>128**	**8**	**>128**	**>128**	0.5	0.25	**>128**	**>128**	>16	NA	2
NDM-7^*d*^	NDM-1 D126N M155L	B	**>128**	**8**	**>128**	**>128**	0.5	0.25	**>128**	**>128**	>16	NA	2
NDM-9^*d*^	NDM-1 E149K	B	**>128**	**>128**	**>128**	**>128**	0.25	0.25	**>128**	**128**	NA	NA	1
NDM-10^*d*^	NDM-1 G63S A68T G209R (R32S G36D)	B	**32**	**2**	**>128**	**>128**	0.5	0.25	**32**	**16**	16	NA	2
NDM-12^*d*^	NDM-1 M155L G235D	B	**>128**	1	**>128**	**>128**	0.25	0.25	**>128**	**>128**	>128	NA	1
NDM-13^*d*^	NDM-1 D89N M155L	B	**>128**	**8**	**>128**	**>128**	0.25	0.25	**>128**	**>128**	>16	NA	1
NDM-15^*d*^	NDM-1 V92L M155L A248V	B	**>128**	**8**	**>128**	**>128**	0.25	0.25	**>128**	**>128**	>16	NA	1
NDM-16b^*d*^	NDM-1 M155L A248V	B	**>128**	**8**	**>128**	**>128**	0.25	0.25	**>128**	**>128**	>16	NA	1
NDM-30^*c*^^*d*^	NDM-1 D236Y	B	**32**	**8**	**>128**	**>128**	0.5	0.25	**>128**	**32**	4	NA	2
VIM-1^*d*^		B	**>128**	**4**	**>128**	**>128**	0.25	0.25	**>128**	**32**	>32	NA	1
VIM-2^*d*^	VIM-1 +17 variants	B	**4**	0.12	**128**	**128**	0.25	0.25	**>128**	**8**	32	1	1
VIM-4^*d*^	VIM-1 S228R	B	**32**	0.25	**>128**	**>128**	0.25	0.25	**>128**	**16**	128	NA	1
VIM-5^*d*^	VIM-1 A130K H224L E225A S228R K308T	B	**4**	0.25	**>128**	**>128**	0.25	0.25	**>128**	**32**	16	NA	1
VIM-7^*d*^	VIM-1 +47 variants	B	**4**	**2**	**64**	**64**	0.25	0.25	**>128**	**32**	2	1	1
VIM-12^*d*^	VIM-1 +7 variants	B	**>128**	**4**	**>128**	**>128**	0.25	0.25	**>128**	**32**	>32	NA	1
VIM-13^*d*^	VIM-1 +19 variants	B	**8**	0.25	**>128**	**>128**	0.5	0.25	**>128**	**8**	32	NA	2
VIM-18^*d*^	VIM-2 Q59R del_147–150	B	**32**	**8**	**>128**	**>128**	0.25	0.25	**>128**	**2**	4	NA	1
VIM-23^*d*^	VIM-2 R228S	B	**64**	1	**>128**	**>128**	0.25	0.25	**>128**	**16**	64	NA	1
VIM-24^*d*^	VIM-2 R228L	B	**64**	1	**>128**	**128**	0.25	0.25	**128**	**8**	64	>1	1
VIM-25^*d*^	VIM-1 +10 variants	B	**4**	0.25	**>128**	**>128**	0.25	0.25	**>128**	**32**	16	NA	1
VIM-26^*d*^	VIM-1 H224L	B	**128**	**4**	**>128**	**>128**	0.25	0.25	**>128**	**32**	32	NA	1
VIM-32^*d*^	VIM-1 E149A	B	**64**	**2**	**>128**	**>128**	0.5	0.25	**>128**	**64**	32	NA	2
VIM-83^*c*^^*d*^	VIM-1 E149K	B	**>128**	**>128**	**>128**	**>128**	0.25	0.25	**>128**	**16**	NA	NA	1
SPM-1^*d*^		B	**64**	**2**	**>128**	**>128**	0.5	0.25	**>128**	**32**	32	NA	2
GIM-1^*d*^		B	**4**	0.25	**>128**	**>128**	0.25	0.25	**>128**	**16**	16	NA	1
SIM-1^*d*^		B	**32**	**64**	**>128**	**>128**	0.5	0.5	**32**	**16**	0.5	NA	1
IMP-1^*d*^		B	**64**	**64**	**>128**	**>128**	0.25	0.25	**32**	**16**	1	NA	1
IMP-4^*d*^	IMP-1 +10 variants	B	**64**	**64**	**>128**	**>128**	0.5	0.25	16	**16**	1	NA	2
IMP-59^*c*^^*d*^	IMP-4 N233Y	B	**128**	**16**	**>128**	**>128**	0.5	0.5	**64**	**32**	8	NA	1
DIM-1^*c*^^*d*^		B	0.25	0.12	**16**	**32**	0.25	0.25	**32**	**0.5**	2	0.5	1
FIM-1^*c*^^*d*^		B	**8**	1	**>128**	**>128**	0.25	0.25	**>128**	**16**	8	NA	1
TMB-1^*c*^^*d*^		B	0.5	0.12	**16**	**16**	0.25	0.12	16	**0.5**	4	1	2
ACT-1^*c*^	ACT-C189 + 44 variants	C	1	0.12	**64**	0.5	**64**	0.5	**32**	0.25	8	128	128
ACT-17	ACT-C189 I16V A88P	C	2	0.25	**>128**	1	**64**	1	**128**	0.06	8	>128	64
ACT-86	ACT-17 del_292–293	C	**128**	**4**	**>128**	**16**	**32**	1	**>128**	≤0.06	32	>8	32
ACT-C189 (P99/AmpC)		C	**4**	0.25	**>128**	1	**64**	1	**>128**	0.12	16	>128	64
ACT-C189 del_292–293	ACT-C189 del_292–293	C	**128**	**8**	**>128**	**8**	**32**	1	**128**	0.06	16	>16	32
ACT-C191	ACT-17 del_289–294 A299V	C	**128**	**8**	**>128**	**16**	**16**	0.5	**128**	0.06	16	>8	32
CMH-3		C	**16**	0.25	**>128**	**128**	**64**	1	**>128**	**4**	64	>1	64
CMH-ENT385	CMH-3 D124N P197S N198H Q245P A280V del_294–295 L296V	C	**64**	1	**>128**	4	**64**	0.5	**128**	0.12	64	>32	128
CMH-ENT385-reversed	CMH-3 D124N P197S N198H Q245P A280V L296V	C	1	0.25	**>128**	1	**64**	1	**64**	0.12	4	>128	64
CMY-2		C	2	0.12	**>128**	1	**64**	0.5	**128**	0.06	16	>128	128
CMY-6	CMY-2 W201L	C	**4**	0.25	**>128**	1	**128**	1	**128**	0.25	16	>128	128
CMY-16	CMY-2 A151S W201R	C	**4**	0.12	**>128**	2	**>128**	1	**>128**	0.25	32	>64	>128
CMY-16 Y150S	CMY-16 Y150S	C	1	0.25	**16**	4	**>128**	**64**	**32**	0.03	4	4	>2
CMY-16 N346H	CMY-16 N346H	C	2	0.25	**>128**	**32**	**>128**	**32**	**64**	0.06	8	>4	>4
CMY-42	CMY-2 V211S	C	**4**	0.25	**>128**	2	**>128**	2	**128**	0.25	16	>64	>64
CMY-172^*c*^	CMY-2 del_290–292 N346I	C	**128**	0.5	**>128**	**128**	**64**	2	**128**	**0.5**	256	>1	32
CMY-185^*c*^	CMY-2 A114E Q120K V211S N346Y	C	**4**	**4**	**>128**	**>128**	**64**	**64**	4	0.03	1	NA	1
DHA-1^*c*^		C	0.12	0.12	**32**	0.5	**4**	0.25	**32**	0.03	1	64	16
FOX-4^*c*^		C	8	0.25	**>128**	**64**	**128**	**4**	**128**	**0.5**	32	>2	32
FOX-5^*c*^	FOX-4 +13 variants	C	0.5	0.12	**32**	1	2	0.25	4	0.03	4	32	8
MIR-17^*c*^		C	0.5	0.25	**64**	0.5	**64**	1	**32**	0.25	2	128	64
MOX-1^*c*^		C	0.5	0.25	**32**	1	**32**	1	16	0.25	2	32	32
MOX-2^*c*^	MOX-1 +26 variants	C	2	0.25	**>128**	**8**	**32**	2	16	**1**	8	>16	16
MOX-9^*c*^	MOX-1 +63 variants	C	4	0.12	**128**	0.5	**16**	0.5	16	0.06	32	256	32
PAC-1		C	128	32	**>128**	**>128**	**32**	**16**	**64**	0.25	4	NA	2
PDC-1		C	2	0.25	**64**	1	**32**	0.5	**>128**	0.12	8	64	64
PDC-3	PDC-1 T79A	C	2	0.25	**64**	1	**32**	0.5	**>128**	0.06	8	64	64
PDC-5	PDC-3 R53Q	C	2	0.25	**64**	1	**32**	0.5	**>128**	0.12	8	64	64
PDC-5 G156D	PDC-5 G156D	C	0.5	0.5	**64**	**8**	**4**	1	16	0.03	1	8	4
PDC-37	PDC-3 G1D V178L V329I G364A	C	1	0.25	**32**	1	**16**	0.5	**>128**	0.12	4	32	32
PDC-50	PDC-3 V211A	C	4	0.25	**>128**	2	**128**	2	**>128**	0.12	16	>64	64
PDC-73	PDC-3 P153L	C	1	0.12	**64**	2	**16**	0.25	**>128**	0.03	8	32	64
PDC-74	PDC-3 G214R	C	4	0.5	**>128**	2	**>128**	**4**	**64**	0.12	8	>64	>32
PDC-75	PDC-3 G214R V329I	C	4	0.25	**>128**	2	**>128**	2	**64**	0.12	16	>64	>64
PDC-80	PDC-1 E219G	C	1	0.25	**64**	1	**8**	0.25	**128**	0.03	4	64	32
PDC-81	PDC-3 P153L G364A	C	4	0.25	**>128**	2	**64**	0.5	**>128**	0.12	16	>64	128
PDC-82	PDC-3 F121L	C	2	0.25	**128**	2	**16**	0.5	**128**	0.06	8	64	32
PDC-86	PDC-37 E219K	C	2	0.25	**>128**	4	**64**	0.5	**64**	0.06	8	>32	128
PDC-87	PDC-37 N346I	C	2	0.25	**>128**	4	**16**	0.5	**64**	0.12	8	>32	32
PDC-88	PDC-37 del_289–290	C	32	0.25	**>128**	1	**16**	0.25	**>128**	0.12	128	>128	64
PDC-89	PDC-37 del_289–291 I329V	C	32	0.5	**32**	2	**16**	0.5	**32**	0.12	64	16	32
PDC-90	PDC-37 del_289–291	C	64	0.5	**>128**	2	**16**	0.5	**64**	0.25	128	>64	32
PDC-91	PDC-37 del_289–292	C	64	0.5	**128**	2	**16**	0.5	**64**	0.06	128	64	32
PDC-92	PDC-37 del_293–294 I329V	C	64	0.5	**>128**	2	**16**	0.5	**128**	0.12	128	>64	32
PDC-221	PDC-1 E219K	C	0.5	0.25	**64**	2	**4**	0.5	8	0.03	2	32	8
PDC-222	PDC-1 T70I	C	1	0.5	**64**	4	**4**	1	16	0.03	2	16	4
PDC-223	PDC-1 del_K204a-222	C	1	0.5	**128**	**8**	**4**	0.5	16	0.03	2	16	8
PDC-237	PDC-3 V19A V211A N346I G364A	C	8	0.25	**>128**	**16**	**64**	1	**64**	0.12	32	>8	64
OXA-1		D (OXA-1)	**32**	**16**	1	0.5	1	0.25	**>128**	0.12	2	2	4
OXA-1 H109Q	OXA-1 H109Q	D (OXA-1-like)	1	0.5	0.5	0.5	0.5	0.25	16	0.03	2	1	2
OXA-31^*c*^	OXA-1 A49V A68P D207E	D (OXA-1-like)	**16**	**8**	0.5	0.5	0.5	0.25	**128**	0.12	2	1	2
OXA-47^*c*^	OXA-1 P35Q T65A T98I K190R E199D D207E N252S	D (OXA-1-like)	**8**	**4**	1	1	2	1	**128**	0.06	2	1	2
OXA-224^*c*^	OXA-1 A49V A53T D207E	D (OXA-1-like)	**16**	**8**	0.5	0.5	0.5	0.25	**>128**	0.06	2	1	2
OXA-2^*c*^		D (OXA-2)	1	0.12	**64**	1	0.5	0.25	8	**1**	8	64	2
OXA-3^*c*^	OXA-2 +26 variants	D (OXA-2-like)	0.25	0.25	4	0.5	0.5	0.25	**>128**	0.03	1	8	2
OXA-32^*c*^	OXA-2 L164I	D (OXA-2-like)	0.12	0.12	2	0.5	0.25	0.25	2	0.03	1	4	1
OXA-34^*c*^	OXA-2 W159C del_258–275	D (OXA-2-like)	**2**	**2**	**128**	**32**	**4**	0.25	8	0.06	1	4	16
OXA-141^*c*^	OXA-2 G162S	D (OXA-2-like)	0.25	0.25	**16**	2	0.5	0.25	4	0.03	1	8	2
OXA-161^*c*^	OXA-2 N148D	D (OXA-2-like)	0.25	0.12	**8**	4	0.25	0.5	4	0.03	2	2	0.5
OXA-210^*c*^	OXA-2 Y158C	D (OXA-2-like)	0.25	0.12	**32**	0.5	0.5	0.12	2	0.016	2	64	4
OXA-5^*c*^		D (OXA-5)	**2**	0.12	1	0.5	**4**	0.25	**128**	0.06	16	2	16
OXA-9^*c*^		D (OXA-9)	**4**	0.25	**8**	0.5	**32**	0.5	**>128**	0.12	16	16	64
OXA-10^*c*^		D (OXA-10)	**8**	0.12	2	1	**32**	**4**	**>128**	0.25	64	2	8
OXA-11^*c*^	OXA-10 N143S G157D	D (OXA-10-like)	**32**	1	**>128**	**>128**	**32**	**4**	**128**	0.12	32	NA	8
OXA-13^*c*^	OXA-10 D55N N73S T107S Y174F G229E S245N E259A	D (OXA-10-like)	**8**	0.12	4	1	**16**	2	**>128**	0.06	64	4	8
OXA-14^*c*^	OXA-10 G157D	D (OXA-10-like)	**16**	0.5	**>128**	**>128**	**64**	**4**	**>128**	0.12	32	NA	16
OXA-16^*c*^	OXA-10 A124T G157D	D (OXA-10-like)	**32**	1	**>128**	**>128**	**32**	2	**64**	0.12	32	NA	16
OXA-17^*c*^	OXA-10 N73S	D (OXA-10-like)	**8**	0.25	4	1	**16**	1	**>128**	0.12	32	4	16
OXA-19^*c*^	OXA-13 S73N G157D	D (OXA-10-like)	**4**	0.12	**>128**	**32**	**16**	2	**128**	0.06	32	>4	8
OXA-28^*c*^	OXA-13 S73N W154G	D (OXA-10-like)	**16**	1	**>128**	**128**	**32**	**4**	**64**	0.12	16	>1	8
OXA-35^*c*^	OXA-13 S73N	D (OXA-10-like)	**8**	0.12	2	1	**32**	**4**	**>128**	**0.5**	64	2	8
OXA-56^*c*^	OXA-13 S27F S73N V89I T230A	D (OXA-10-like)	**8**	0.25	2	1	**32**	**8**	**>128**	**0.5**	32	2	4
OXA-74^*c*^	OXA-10 A66V N73S	D (OXA-10-like)	**16**	0.25	**8**	2	**64**	**8**	**>128**	**0.5**	64	4	8
OXA-142^*c*^	OXA-10 N73S G157D	D (OXA-10-like)	**16**	0.5	**>128**	**128**	**16**	2	**128**	0.12	32	>1	8
OXA-145^*c*^	OXA-13 S73N del_155	D (OXA-10-like)	**16**	0.5	**>128**	**>128**	**16**	2	**64**	0.12	32	NA	8
OXA-147^*c*^	OXA-13 S73N W154L	D (OXA-10-like)	0.5	0.12	**16**	4	2	0.5	4	0.016	4	4	4
OXA-183^*c*^	OXA-19 I150T	D (OXA-10-like)	**16**	0.5	**>128**	**>128**	**32**	2	**64**	0.12	32	NA	16
OXA-240^*c*^	OXA-10 K100N	D (OXA-10-like)	**8**	0.25	2	1	**32**	**4**	**>128**	**0.5**	32	2	8
OXA-256^*c*^	OXA-10 N73D	D (OXA-10-like)	**8**	0.25	**8**	2	**16**	2	**128**	0.25	32	4	8
OXA-18^*c*^		D (OXA-18)	**128**	1	**>128**	2	**>128**	**4**	**>128**	**0.5**	128	>64	>32
OXA-23^*d*^		D (OXA-23)	**8**	**4**	0.5	0.5	0.5	0.25	**>128**	2	2	1	2
OXA-57^*c*^		D (OXA-42-like)	0.12	0.12	0.5	0.5	0.5	0.25	**64**	0.06	1	1	2
OXA-59	OXA-57 D170N	D (OXA-42-like)	0.25	0.25	0.5	0.5	0.5	0.5	**>128**	0.25	1	1	1
OXA-46^*c*^		D (OXA-46)	0.5	0.12	**16**	0.5	0.5	0.25	**64**	0.12	4	32	2
OXA-48^*d*^		D (OXA-48)	2	0.25	1	0.5	0.5	0.5	**>128**	**4**	8	2	1
OXA-162^*d*^	OXA-48 T213A	D (OXA-48-like)	**4**	0.25	2	0.5	0.5	0.25	**>128**	**2**	16	4	2
OXA-163	OXA-48 S212D del_214–217	D (OXA-48-like)	**64**	0.25	**>128**	4	**>128**	1	**>128**	**0.5**	256	>32	>128
OXA-181^*d*^	OXA-48 T104A N110D E168Q S171A	D (OXA-48-like)	2	0.25	1	0.5	0.5	0.25	**>128**	**2**	8	2	2
OXA-204^*d*^	OXA-48 Q98H T99R	D (OXA-48-like)	1	0.12	1	0.5	0.25	0.25	**>128**	**1**	8	2	1
OXA-232^*d*^	OXA-181 R214S	D (OXA-48-like)	2	0.25	1	0.5	0.5	0.25	**>128**	**2**	8	2	2
OXA-244^*d*^	OXA-48 R214G	D (OXA-48-like)	1	0.25	2	0.5	1	0.25	**>128**	**2**	4	4	4
OXA-245^*d*^	OXA-48 E125Y	D (OXA-48-like)	1	0.12	1	0.5	0.5	0.25	**>128**	**2**	8	2	2
OXA-247^*c*^^*d*^	OXA-48 Y211S S212N del_214–217	D (OXA-48-like)	0.25	0.12	2	0.5	**4**	0.5	**>128**	0.06	2	4	8
OXA-370^*c*^	OXA-48 S212E	D (OXA-48-like)	0.25	0.25	0.5	0.25	0.5	0.12	**64**	0.25	1	2	4
OXA-405^*c*^	OXA-48 del_213–216	D (OXA-48-like)	**8**	0.12	**64**	1	**16**	0.5	**>128**	0.12	64	64	32
OXA-436^*c*^^*d*^	OXA-181 + 13 variants	D (OXA-48-like)	0.25	0.12	0.5	0.5	0.25	0.25	**>128**	**2**	2	1	1
OXA-484^*d*^	OXA-181 R214G	D (OXA-48-like)	2	0.25	4	1	1	0.25	**>128**	**2**	8	4	4
OXA-517^*c*^^*d*^	OXA-48 R214K del_215–216	D (OXA-48-like)	**16**	0.25	**128**	2	**16**	0.5	**>128**	**2**	64	64	32
OXA-519^*c*^^*d*^	OXA-48 V120L	D (OXA-48-like)	0.12	0.12	0.5	0.5	0.25	0.25	**64**	**4**	1	1	1
OXA-50		D (OXA-50)	0.25	0.25	0.5	0.5	0.5	0.25	4	0.12	1	1	2
OXA-396	OXA-50 D109E R167H	D (OXA-50-like)	0.25	0.25	1	0.5	**4**	1	4	0.25	1	2	4
OXA-51^*d*^		D (OXA-51)	0.25	0.12	0.5	0.5	0.25	0.25	**64**	**0.5**	2	1	1
OXA-66^*d*^	OXA-51 E36V V48A Q107K	D (OXA-51-like)	0.25	0.25	1	1	0.25	0.25	**32**	0.25	1	1	1
OXA-58^*d*^		D (OXA-58)	0.25	0.25	1	0.5	0.25	0.25	**>128**	**0.5**	1	2	1
OXA-427^*c*^^*d*^		D (OXA-427)	**8**	0.25	**>128**	**8**	**64**	2	**>128**	**0.5**	32	>16	32
LCR-1^*c*^		D	0.25	0.25	1	1	1	0.5	16	0.12	1	1	2

^*a*^ β-lactamases were produced from either pTU501 or pTU672. The MICs shown are modal values (µg/mL) determined from at least three experiments. The MIC values that increased ≥16-fold relative to the vector control are shown in bold. Taniborbactam and avibactam were tested in combination with cefepime and ceftazidime at a fixed concentration of 4 μg/mL each (26). Routine QC isolates *E. coli* ATCC 25922, *E. coli* NCTC 13353 and *K. pneumoniae* ATCC 700603 were used to confirm that MICs of antibiotics were within acceptable QC ranges (27) in each experiment.

^*b*^ ATM, aztreonam; AVI, avibactam; CAZ, ceftazidime; FEP, cefepime; MEM, meropenem; NA, not applicable; PIP, piperacillin; TAN, taniborbactam.

^*c*^ β-lactamases produced from pTU672.

^*d*^ β-lactamases reported as carbapenemases (KPCs, certain GES variants, SME, OXA-48 types, OXA-51 types, MBLs) at Beta-Lactamase DataBase (25) and in literature.

^*e*^ Variants in residues are shown using the standard numbering schemes for Class A, B, and C (28, 29, 30). The actual residue numbers are used to show variants in Class D β-lactamases because a standard numbering scheme has not been established yet.

**TABLE 2.** MIC_50_, MIC_90_, and MIC distributions against *E. coli* isogenic strains overproducing β-lactamases of all classes of β-lactamases (n = 190)^*a*^

Compound	MIC_50_ (µg/mL)	MIC_90_ (µg/mL)	MIC (µg/mL)											
			0.12	0.25	0.5	1	2	4	8	16	32	64	128	>128
FEP	8	128	4	17	10	15	18	22	22	17	16	16	**18**	15
		Cumulative %	2.1	11.1	16.3	24.2	33.7	45.3	56.8	65.8	74.2	82.6	**92.1**	100.0
FEP-TAN	0.25	8	34	82	23	14	9	8	**12**	2	1	3		2
		Cumulative %	17.9	61.1	73.2	80.5	85.3	89.5	**95.8**	96.8	97.4	98.9		100.0
CAZ	>128	>128			11	13	8	6	5	9	7	21	11	**99**
		Cumulative %			5.8	12.6	16.8	20.0	22.6	27.4	31.1	42.1	47.9	**100.0**
CAZ-AVI	4	>128		1	34	30	28	16	9	8	5	6	11	**42**
		Cumulative %		0.5	18.4	34.2	48.9	57.4	62.1	66.3	68.9	72.1	77.9	**100.0**
ATM	16	>128		31	32	5	5	10	4	26	20	20	7	**30**
		Cumulative %		16.3	33.2	35.8	38.4	43.7	45.8	59.5	70.0	80.5	84.2	**100.0**
ATM-AVI	0.5	4	3	73	39	28	17	**12**	4	4	3	5		2
		Cumulative %	1.6	40.0	60.5	75.3	84.2	**90.5**	92.6	94.7	96.3	98.9		100.0

^*a*^ ATM, aztreonam; AVI, avibactam; CAZ, ceftazidime, FEP, cefepime; TAN, taniborbactam. The MIC_50_s are underlined, and MIC_90_s are shown in bold. The MIC_50_ and MIC_90_ values (also in Table 3) are used to compare the activity of agents against the isogenic strains overproducing β-lactamases and are not relevant to the susceptibility of clinical isolates or a clinical evaluation.

**TABLE 3:** MIC_50_, MIC_90_, and MIC distributions against *E. coli* isogenic strains overproducing β-lactamases by Ambler class^*a*^

Compound	MIC_50_ (µg/mL)	MIC_90_ (µg/mL)	MIC (µg/mL)												
			0.12	0.25	0.5	1	2	4	8	16	32	64	128	>128	
Class A β-lactamases (*n* = 50)															
FEP	16	128		2	2	2	2	4	7	7	6	5	**9**	4	
		Cumulative %		4.0	8.0	12.0	16.0	24.0	38.0	52.0	64.0	74.0	**92.0**	100.0	
FEP-TAN	0.25	1	5	27	9	**5**	3	1							
		Cumulative %	10.0	64.0	82.0	**92.0**	98.0	100.0							
CAZ	>128	>128				1		2	1	3	1	8	4	**30**	
		Cumulative %				2.0		6.0	8.0	14.0	16.0	32.0	40.0	**100.0**	
CAZ-AVI	4	>128			3	5	10	8	4	4	1	4	5	**6**	
		Cumulative %			6.0	16.0	36.0	52.0	60.0	68.0	70.0	78.0	88.0	**100.0**	
ATM	128	>128			3	1	2	1	3	7	3	4	4	**22**	
		Cumulative %			6.0	8.0	12.0	14.0	20.0	34.0	40.0	48.0	56.0	**100.0**	
ATM-AVI	1	32		11	9	11	4	3	2	3	**2**	3		2	
		Cumulative %		22.0	40.0	62.0	70.0	76.0	80.0	86.0	**90.0**	96.0		100.0	
Class B β-lactamases (*n* = 34)															
FEP	64	>128		1	1			5	2		5	6	3	**11**	
		Cumulative %		2.9	5.9			20.6	26.5		41.2	58.8	67.6	**100.0**	
FEP-TAN	2	64	3	5		4	5	3	8	1		**3**		2	
		Cumulative %	8.8	23.5		35.3	50.0	58.8	82.4	85.3		**94.1**		100.0	
CAZ	>128	>128								2		1	1	**30**	
		Cumulative %								5.9		8.8	11.8	**100.0**	
CAZ-AVI	>128	>128								1	1	1	2	**29**	
		Cumulative %								2.9	5.9	8.8	14.7	**100.0**	
ATM	0.25	0.5		23	**11**										
		Cumulative %		67.6	**100.0**										
ATM-AVI	0.25	0.25	1	**31**	2										
		Cumulative %	2.9	**94.1**	100.0										
Class C β-lactamases (*n* = 48)															
FEP	2	128	1		5	8	10	10	2	1	2	4	**5**		
		Cumulative %	2.1		12.5	29.2	50.0	70.8	75.0	77.1	81.3	89.6	**100.0**		
FEP-TAN	0.25	4	7	26	9	1		**2**	2		1				
		Cumulative %	14.6	68.8	87.5	89.6		**93.8**	97.9		100.0				
CAZ	>128	>128								1	5	10	4	**28**	
		Cumulative %								2.1	12.5	33.3	41.7	**100.0**	
CAZ-AVI	2	64			4	13	13	5	4	3	1	**1**	2	2	
		Cumulative %			8.3	35.4	62.5	72.9	81.3	87.5	89.6	**91.7**	95.8	100.0	
ATM	32	>128					1	5	1	11	8	13	3	**6**	
		Cumulative %					2.1	12.5	14.6	37.5	54.2	81.3	87.5	**100.0**	
ATM-AVI	0.5	4		5	19	13	5	**2**		1	1	2			
		Cumulative %		10.4	50.0	77.1	87.5	**91.7**		93.8	95.8	100.0			
Class D β-lactamases (*n* = 58)															
FEP	2	16	3	14	2	5	6	3	11	**9**	3	1	1		
		Cumulative %	5.2	29.3	32.8	41.4	51.7	56.9	75.9	**91.4**	96.6	98.3	100.0		
FEP-TAN	0.25	2	19	24	5	4	**1**	2	2	1					
		Cumulative %	32.8	74.1	82.8	89.7	**91.4**	94.8	98.3	100.0					
CAZ	8	>128			11	12	8	4	4	3	1	2	2	**11**	
		Cumulative %			19.0	39.7	53.4	60.3	67.2	72.4	74.1	77.6	81.0	**100.0**	
CAZ-AVI	1	128		1	27	12	5	3	1		2		**2**	5	
		Cumulative %		1.7	48.3	69.0	77.6	82.8	84.5		87.9		**91.4**	100.0	
ATM	1	32		8	18	4	2	4		8	**9**	3		2	
		Cumulative %		13.8	44.8	51.7	55.2	62.1		75.9	**91.4**	96.6		100.0	
ATM-AVI	0.5	4	2	26	9	4	8	**7**	2						
		Cumulative %	3.4	48.3	63.8	70.7	84.5	**96.6**	100.0						

^*a*^ ATM, aztreonam; AVI, avibactam; CAZ, ceftazidime; FEP, cefepime; TAN, taniborbactam.The MIC_50_s are underlined, and MIC_90_s are bolded.

Against all β-lactamase-overproducing strains (*n* = 190), the cefepime MIC_50_ and MIC_90_ were 8 and 128 µg/mL, respectively. The cefepime MIC was elevated to ≥16 µg/mL in 82 strains (43.2%); 17 strains showed an MIC of 16 µg/mL, and 65 strains showed an MIC of ≥32 µg/mL (Table 2). The addition of 4 µg/mL taniborbactam restored the cefepime MIC to ≤8 µg/mL and ≤16 µg/mL for 182 strains (95.8%) and 184 strains (96.8%), respectively. The cefepime-taniborbactam MIC_50_ and MIC_90_ were 0.25 and 8 µg/mL, respectively, demonstrating ≥16-fold potentiation of cefepime activity by taniborbactam. The cefepime-taniborbactam MICs were distributed as follows: two strains at >128 µg/mL (NDM-9 and VIM-83), three strains at 64 µg/mL (SIM-1, IMP-1, and IMP-4), one strain at 32 µg/mL (PAC-1), two strains at 16 µg/mL (IMP-59, OXA-1), 12 strains at 8 µg/mL (seven NDM variants, VIM-18, two ACT variants, and two OXA-1 variants), eight strains at 4 µg/mL (VEB-14, three VIM, one ACT-17 variant, CMY-185, one OXA-1 variant [OXA-47], and OXA-23), and 162 strains at ≤2 µg/mL.

The ceftazidime MIC_50_ and MIC_90_ were both >128 µg/mL. The ceftazidime MIC was elevated to ≥16 µg/mL in 147 strains. The addition of 4 µg/mL avibactam restored the ceftazidime MIC to ≤8 µg/mL for 118 strains (62.1%) and ≤16 µg/mL for 126 strains (66.3%) (Table 2). The ceftazidime-avibactam MIC_50_ and MIC_90_ were 4 and >128 µg/mL, respectively, which are ≥16-fold higher than those for cefepime-taniborbactam. The aztreonam MIC_50_ and MIC_90_ were 16 and >128 µg/mL, respectively. The aztreonam MIC was elevated to ≥8 µg/mL in 107 strains. The addition of 4 µg/mL avibactam restored the aztreonam MIC to ≤4 µg/mL for 172 strains (90.5%) and to ≤8 µg/mL for 176 strains (92.6%). The aztreonam-avibactam MIC_50_ and MIC_90_ were 0.5 and 4 µg/mL, respectively, which are within 2-fold of those for cefepime-taniborbactam.

Excluding class B metallo-β-lactamases that were inhibited only by taniborbactam and did not inactivate aztreonam, MIC_50_/MIC_90_ values against 156 strains producing serine-β-lactamases were 4/128 µg/mL for cefepime alone, 0.25/1 µg/mL for cefepime-taniborbactam, 128/>128 µg/mL for ceftazidime alone, 2/128 µg/mL for ceftazidime-avibactam, 16/>128 µg/mL for aztreonam alone, and 0.5/8 µg/mL for aztreonam-avibactam.

A ≥16-fold increase in MIC compared with the respective vector control strain was shown in 67.9% (129/190) of the strains for cefepime and 19.5% (37/190) of the strains for cefepime-taniborbactam (Table 4). Taniborbactam decreased the cefepime MIC ≥8-fold for 113 enzymes (87.6%; 42 Ambler class A, 24 class B, 23 class C, and 24 class D) among the 129 β-lactamases that increased the cefepime MIC ≥16-fold (42 Ambler class A, 32 B, 25 C, and 30 D). For ceftazidime and ceftazidime-avibactam, 80.0% (152/190) and 42.6% (81/190) of strains, respectively, showed a ≥16-fold increase in MIC. Avibactam decreased the ceftazidime MIC ≥8-fold for 80 enzymes (52.6%; 29 Ambler class A, 41 C, and 10 D) among the 152 β-lactamases that increased the ceftazidime MIC ≥16-fold (47 Ambler class A, 34 B, 48 C, and 23 D). For aztreonam and aztreonam-avibactam, 61.6% (117/190) and 53.7% (102/190) of the strains, respectively, showed a ≥16-fold increase in MIC. None of the 34 class B metallo-β-lactamases tested in this study (all members of subclass B1) increased the aztreonam MIC >2-fold, consistent with the known stability of aztreonam to metallo-β-lactamases (31, 32). Avibactam decreased the aztreonam MIC ≥8-fold for 102 enzymes (87.2%; 36 Ambler class A, 42 C, and 24 D) among the 117 β-lactamases that increased the aztreonam MIC ≥16-fold (44 class A, 47 C, and 26 D).

**TABLE 4.** The number of β-lactamases inhibited by taniborbactam and avibactam^*b*^

Description	Total (*n* = 190)	Class A (*n* = 50)	Class B (*n* = 34)	Class C (*n* = 48)	Class D (*n* = 58)
FEP MIC increase ≥16-fold	129	42	32	25	30
^*a*^FEP MIC ≥8-fold decrease by TAN	113	42	24	23	24
CAZ MIC increase ≥16-fold	152	47	34	48	23
^*a*^CAZ MIC ≥8-fold decrease by AVI	80	29	0	41	10
ATM MIC increase ≥16-fold	117	44	0	47	26
^*a*^ATM MIC ≥8-fold decrease by AVI	102	36	NA	42	24

^*a*^ Among the β-lactamases that increased MIC of FEP, CAZ, and ATM ≥16-fold relative to MIC against the vector control, β-lactamases with ≥8-fold decrease of FEP MIC by TAN, CAZ MIC by AVI, and ATM MIC by AVI, respectively, are counted. The fold-decrease in MIC of FEP by TAN, CAZ by AVI, and ATM by AVI is shown in Table 1.

^*b*^ ATM, aztreonam; AVI, avibactam; CAZ, ceftazidime; FEP, cefepime; NA, not applicable; TAN, taniborbactam.

These data highlight two key advantages of cefepime-taniborbactam relative to ceftazidime-avibactam: first, a wider range of β-lactamases negatively impacted ceftazidime than cefepime, and second, taniborbactam provides a broader spectrum of inhibitory activity by the cefepime-taniborbactam combination relative to avibactam for the ceftazidime-avibactam combination. Compared with aztreonam-avibactam, cefepime-taniborbactam shows a similar coverage of these β-lactamases.

### Class A serine-β-lactamase coverage by cefepime-taniborbactam

Of the 50 isogenic strains overproducing Ambler class A β-lactamases, 31 strains (62.0%) showed an MIC of ≥16 µg/mL to cefepime alone; seven strains showed an MIC of 16 µg/mL, six strains showed an MIC of 32 µg/mL, and 18 strains showed an MIC of ≥64 µg/mL (Table 3). Upon the addition of taniborbactam, the cefepime-taniborbactam MIC against all strains decreased to ≤4 µg/mL. The cefepime MIC_90_ decreased from 128 µg/mL to 1 µg/mL with the inclusion of taniborbactam, demonstrating potent inhibitory activity of the β-lactamase inhibitor against Ambler class A β-lactamases. For ceftazidime and ceftazidime-avibactam, 46 strains (92.0%) and 20 strains (40.0%) showed an MIC of ≥16 µg/mL, respectively. For aztreonam and aztreonam-avibactam, 43 strains (86.0%) and 12 strains (24.0%) showed an MIC of ≥8 µg/mL, respectively.

The 20 problematic class A β-lactamases impacting ceftazidime-avibactam are nine individual KPC variants, six PER variants, two GES variants, and three VEB variants (Table 1). These KPC variants had either a D179Y substitution in the Ω loop (residues 164–179), internal deletions or a substitution in the 237–243 loop that borders the active site pocket, or insertions at residue 269 located in loop 267–275. These variants have been implicated in reduced susceptibility to ceftazidime-avibactam (33–39). Production of PER and VEB (ESBLs in Enterobacterales and *P. aeruginosa*) decreased susceptibility to ceftazidime-avibactam, consistent with previous reports (40–43). GES-6 and GES-13, both of which had the E104K variant, are also associated with elevated MIC of ceftazidime-avibactam (44). The 12 class A β-lactamases impacting aztreonam-avibactam were six PER variants, two VEB variants, two KPC variants, NmcA, and SME-2 (Table 1). Every PER β-lactamase tested herein inactivated aztreonam as well as ceftazidime and was not sufficiently inhibited by avibactam to restore susceptibility. VEB-25 and KPC-87 that mediate resistance to ceftazidime-avibactam (36, 42, 43) also impacted aztreonam-avibactam. Importantly, none of the class A β-lactamases that reduced susceptibility to ceftazidime-avibactam and aztreonam-avibactam increased cefepime-taniborbactam MIC to ≥8 µg/mL.

### Class B metallo-β-lactamase coverage by cefepime-taniborbactam

Of the 34 isogenic strains overproducing Ambler class B metallo-β-lactamases (MBLs), 25 strains (73.5%) showed an MIC of ≥16 µg/mL to cefepime alone; five strains showed an MIC of 32 µg/mL, and 20 strains showed an MIC of ≥64 µg/mL (Table 3). Upon addition of taniborbactam, 28 strains showed a cefepime-taniborbactam MIC of ≤8 µg/mL and six strains overproducing NDM-9, VIM-83, SIM-1, and three IMP enzymes showed a cefepime-taniborbactam MIC of ≥16 µg/mL. The geometric mean value of cefepime MICs was reduced by taniborbactam from 33.3 µg/mL to 2.9 µg/mL, demonstrating the potent *in vitro* inhibitory activity of taniborbactam against MBLs. Notably, the antibacterial activity of cefepime against strains overproducing prevalent NDM enzymes (NDM-1, NDM-5, and NDM-7) was potentiated >16-fold by the addition of taniborbactam (Table S1). By contrast, for ceftazidime, all strains (100%) showed an MIC of ≥16 µg/mL, and the addition of avibactam failed to potentiate ceftazidime antibacterial activity, consistent with the observation that avibactam lacks MBL-inhibitory activity (45). For aztreonam, all strains (100%) showed an MIC of ≤0.5 µg/mL, consistent with the lack of MBL hydrolytic activity against aztreonam (31, 32).

Despite the potent activity against NDM β-lactamases (e.g., NDM-1, NDM-5, and NDM-7) and VIM-2-like enzymes, taniborbactam did not sufficiently inhibit IMP-1, two NDM variants (NDM-9 and NDM-30), and VIM-1-like β-lactamases such as VIM-83, as described previously (2, 46–51). NDM-9 and VIM-83 have a single amino acid substitution Glu149Lys (hereinafter residue number referring to the standard numbering scheme [28]) that impacts a key salt bridge between taniborbactam and the protein (46, 52). Among 20,725 Enterobacterales and 7,919 *P. aeruginosa* clinical isolates tested for cefepime-taniborbactam activity in the GEARS global surveillance program, only one isolate carried NDM-9, whereas neither NDM-30 nor VIM-83 was identified (8).

Taniborbactam inhibited SPM-1, GIM-1, and FIM-1, but not SIM-1, IMP-1, or IMP-4 (Table 1). Interestingly, IMP-59 was found in a clinical isolate of *E. coli* (IHMA 1468388) where the addition of 4 µg/mL taniborbactam decreased the cefepime MIC from 16 µg/mL to 0.12 µg/mL (data not shown). In the isogenic background, taniborbactam reduced the cefepime MIC of the strain overproducing IMP-59 by 8-fold, indicating the inhibition of IMP-59 by taniborbactam (Table 1). IMP-59 has a single amino acid substitution, Asn233Tyr, from IMP-4. Furthermore, taniborbactam inhibited an engineered IMP-1 variant where the same substitution was introduced (Table S4), indicating that Asn233 is a key residue for IMP insensitivity to inhibition by taniborbactam. Interestingly, another boronate inhibitor ledaborbactam (formerly VNRX-5236) (53, 54) inhibited the IMP Asn233Tyr variants, whereas neither avibactam nor vaborbactam (13) inhibited the variants (Table S4). Asn233 is a well-conserved residue located in the L3 loop of the MBL active sites and contributes to substrate binding (55). A homology model of IMP-4, based on the X-ray structure of IMP-1 (PDB 5EV6) (56), showed high conservation of residues within the active sites, whereas a homology model of IMP-59 indicates that Tyr233 creates an extended lipophilic surface within the active site (Fig. S2). A docking structure of the IMP-59 homology model in complex with taniborbactam showed that Tyr233 provided extensive hydrophobic interactions with the cyclohexyl-acetyl chain of taniborbactam (Fig. S3), which could explain why taniborbactam inhibits IMP-59 but not IMP-4.

### Class C serine-β-lactamase coverage by cefepime-taniborbactam

Of the 48 isogenic strains overproducing Ambler class C β-lactamases, 12 strains (25.0%) showed an MIC of ≥16 µg/mL to cefepime alone; one strain showed an MIC of 16 µg/mL, two showed an MIC of 32 µg/mL, and nine showed an MIC of ≥64 µg/mL (Table 3). Upon the addition of taniborbactam, cefepime MICs against all strains were reduced to ≤8 µg/mL except for PAC-1. The cefepime MIC_90_ decreased from 128 µg/mL to 4 µg/mL by the addition of taniborbactam, demonstrating potent *in vitro* inhibitory activity of taniborbactam against Ambler class C β-lactamases. By contrast, for ceftazidime, 100% of the strains showed an MIC of ≥16 µg/mL, and upon the addition of avibactam, nine strains (18.8%) showed an MIC of ≥16 µg/mL. For aztreonam and aztreonam-avibactam, 42 strains (87.5%) and four strains (8.3%) showed an MIC of ≥8 µg/mL, respectively.

Deletions of residues in the H-10 helix in class C enzymes are associated with resistance to cephalosporins including cefepime and cefiderocol as well as ceftazidime-avibactam (57–61). Consistently, strains overproducing ACT-86, ACT-C189_Δ292–293, CHM-ENT385, CMY-172, and PDC variants (PDC-88, -89, -90, -91, and -92), which all had a deletion in the H-10 helix, showed elevated cefepime MIC. The addition of taniborbactam restored cefepime activity against these strains, suggesting that deletions of the H-10 helix do not impact the inhibitory activity of taniborbactam against class C β-lactamases.

Substitutions of Asn346 of CMY enzymes are associated with resistance to aztreonam-avibactam and ceftazidime-avibactam (62–64). In addition, FOX-4, identified in a ceftazidime-avibactam-resistant *P. aeruginosa* clinical isolate, has Ile346 instead of Asn346, which contributes to substrate binding (65, 66). Consistently, strains overproducing CMY-172 (which also has a deletion in the H-10 helix), CMY-185, and FOX-4, which all have a substitution of Asn346, showed high MICs of ceftazidime-avibactam and/or aztreonam-avibactam (Table 1). In contrast, cefepime and cefepime-taniborbactam MICs were 128 and 0.5 µg/mL, respectively, against the strain producing CMY-172, demonstrating potent inhibition by taniborbactam. Against the strain producing CMY-185, MICs of cefepime and cefepime-taniborbactam were both 4 µg/mL, indicating that CMY-185 is not an efficient cefepimase, although it is not inhibited by taniborbactam. Cefepime-taniborbactam retained substantial activity against this strain overproducing FOX-4 (MIC of 0.25 µg/mL) (Table 1). Cefepime-taniborbactam MICs were ≤0.5 µg/mL against all 22 strains overproducing PDC enzymes including two variants having an Asn346Ile substitution (PDC-87 and PDC-237).

Finally, PAC-1 is a poorly characterized β-lactamase that has been reported in only four *P. aeruginosa* clinical isolates resistant to ceftazidime-avibactam (67). The isogenic strain producing PAC-1 showed MICs of cefepime-taniborbactam, ceftazidime-avibactam, and aztreonam-avibactam at 32, >128, and 16 µg/mL, respectively. This enzyme, which is rarely found in clinical isolates, is the only one that showed a high cefepime-taniborbactam MIC among 48 class C β-lactamases tested in this study.

### Class D serine-β-lactamase coverage by cefepime-taniborbactam

Of the 58 isogenic strains overproducing Ambler class D β-lactamases (including 17 OXA-10-like, 15 OXA-48-like, seven OXA-2-like, and five OXA-1-like enzymes), 14 strains (24.1%) showed an MIC of ≥16 µg/mL to cefepime alone; nine strains showed an MIC of 16 µg/mL, three strains showed an MIC of 32 µg/mL, and two strains showed an MIC of ≥64 µg/mL (Table 3). Upon the addition of taniborbactam, one strain overproducing OXA-1 showed a cefepime MIC of 16 µg/mL, and all other strains including all carbapenem-hydrolyzing OXA enzymes had cefepime-taniborbactam MICs of ≤8 µg/mL. The cefepime MIC_90_ was decreased by taniborbactam from 16 µg/mL to 2 µg/mL, demonstrating the potent *in vitro* inhibitory activity of taniborbactam against Ambler class D β-lactamases. By contrast, for ceftazidime, 19 strains (32.8%) showed an MIC of ≥16 µg/mL, and upon the addition of avibactam, nine (15.5%) still showed an MIC of ≥16 µg/mL. For aztreonam, 22 strains (37.9%) showed an MIC of ≥16 µg/mL, and upon the addition of avibactam, every strain showed an MIC of ≤8 µg/mL.

OXA-1 mediates reduced susceptibility to cefepime, but not to ceftazidime (68, 69). Among the OXA-overproducing strains, those producing OXA-1 type enzymes (OXA-1, OXA-31, OXA-47, and OXA-224) as well as OXA-23 showed the highest MIC increases for cefepime-taniborbactam relative to the vector control strain (Table S1). OXA-23 is found rarely in Enterobacterales, whereas it is more prevalent in *Acinetobacter baumannii* (70–72). Taniborbactam potentiated cefepime activity only 2-fold against isogenic strains overproducing these OXA-1-like enzymes and OXA-23. Taniborbactam did not reduce the cefepime MIC against the OXA-2-like OXA-34-overproducing strain (cefepime-taniborbactam MIC, 2 µg/mL). For all other strains overproducing OXA β-lactamases including OXA-10 and carbapenemases such as OXA-48 type enzymes, cefepime-taniborbactam MICs were ≤1 µg/mL (Table 1).

Although ceftazidime-avibactam MIC was low at ≤1 µg/mL against strains producing most OXA β-lactamases including OXA-1 type and OXA-48 enzymes, it was ≥128 µg/mL against strains producing seven OXA-10 type β-lactamases (OXA-11,–14, -16,–28, -142,–145, and −183) and 32 µg/mL against OXA-34 (OXA-2-like) and OXA-19 (OXA-10-like) β-lactamases. This is consistent with a previous report that OXA-10 variants mediated resistance to ceftazidime-avibactam (73). In contrast, none of the OXA-overproducing strains showed an aztreonam-avibactam MIC of ≥16 µg/mL. Two strains overproducing OXA-10 variants (OXA-56 and OXA-74) showed an aztreonam-avibactam MIC of 8 µg/mL. From this side-by-side comparison within class D β-lactamase overproducers, cefepime-taniborbactam, ceftazidime-avibactam, and aztreonam-avibactam generally cover these class D enzymes, with cefepime-taniborbactam exhibiting increased potency compared with ceftazidime-avibactam against OXA-10 type enzymes, whereas ceftazidime-avibactam has greater potency against OXA-1 type enzymes.

### Conclusions

Overall, the data provided here enriched the understanding of the therapeutic spectrum of cefepime-taniborbactam compared with other agents against the extensive range of β-lactamases representative of different classes. Within this panel of 190 distinct β-lactamase-overproducing strains, MIC_50_/MIC_90_ values were 8/128 µg/mL for cefepime, 0.25/8 µg/mL for cefepime-taniborbactam, >128/>128 µg/mL for ceftazidime, 2/>128 µg/mL for ceftazidime-avibactam, 16/>128 µg/mL for aztreonam, and 0.5/4 µg/mL for aztreonam-avibactam (Table 2). The effects of permeability and efflux on the β-lactam and β-lactamase inhibitor and variants of β-lactam-targeting PBPs as well as any convoluting effects of multiple β-lactamases in clinical isolates were not evaluated in this study. However, these data highlight that the spectrum of β-lactamase coverage of cefepime-taniborbactam is broader than ceftazidime-avibactam and comparable with aztreonam-avibactam against these 190 isogenic strains producing β-lactamases found in Enterobacterales and *P. aeruginosa*.

## MATERIALS AND METHODS

### Antibiotics

Cefepime (catalog number: 1097636), meropenem (catalog number: 1392454), and aztreonam (catalog number: 1046205) were purchased from USP (Rockville, MD). Ceftazidime (catalog number: A6987-1G) and piperacillin (catalog number: P8396-1G) were purchased from Sigma (St. Louis, MO). Taniborbactam was synthesized by chemists at Venatorx. Avibactam was prepared as described previously (2).

### Construction of isogenic strains of *E. coli* producing individual β-lactamases

The isogenic strains were used to establish the spectrum of inhibitory activity against 190 β-lactamases engineered to be overproduced in the *E. coli* K-12 DH10B strain. DH10B carries chromosomal AmpC (ESC-1), a narrow-spectrum cephalosporinase, but the strain is inherently susceptible to β-lactams because the expression of ESC-1 is at a low level and is not inducible (74). To maximize the expression levels of β-lactamases, plasmids were designed to have an origin of replication with a high copy number (*pBR* origin), the *bla*_TEM-1_ promoter (the *P3* promoter that supports the constitutive expression of TEM-1 [75]), the DNA sequence encoding the signal sequence of TEM-1, and the codon-optimized sequence encoding the mature and active portion of each β-lactamase. For β-lactamases that are shown or predicted to be lipoprotein (i.e., NDMs), periplasmic active portions of the enzymes were expressed in the periplasm. Plasmids were created using DNA synthesis and cloning into pTwist Chlor High Copy using the *E. coli* K-12 strain DH10B, and the entire sequence of each constructed plasmid was verified by Next-Generation Sequencing at Twist Bioscience (South San Francisco, CA). In the DH10B strains carrying the plasmids, β-lactamases were expressed constitutively at high levels in the periplasm using the *bla*_TEM-1_ promoter and signal sequence. The complete DNA sequence of the control expression plasmid pTU501 that expresses only the signal peptide is available at GenBank (accession number MN307371.1). The other control plasmid pTU672 (GenBank accession number PQ862157) was constructed based on pTU501 using gene synthesis with the addition of the *Pseudomonas* replication origin (*ori^1600^*) and *aacC3,* coding for an aminoglycoside acetyltransferase that provides gentamicin-resistance selection (Fig. S1). Both pTU501 and pTU672 carried *cat*, the gene coding for chloramphenicol acetyltransferase providing selection with chloramphenicol. pTU501 was initially used for cloning, and after pTU672 was constructed, it was prioritized as a vector for cloning. The protein sequences for each of the β-lactamases were obtained from the Beta-Lactamase DataBase (http://www.bldb.eu/) (25). The individual β-lactamase signal peptides were identified by literature search or using SignalP 4.1 (76). The identification of the signal sequence enabled the assignment of the mature, active β-lactamase sequence to be appended to the TEM-1 signal sequence. Expression of each β-lactamase was confirmed by elevated MICs of control β-lactam antibiotics known to be substrates of each specific β-lactamase. The *E. coli* DH10B strains carrying pTU501 or pTU672 expressing only the TEM-1 signal sequence from the *bla*_TEM-1_ promoter were used as controls to determine the baseline MIC in the absence of expression of an active β-lactamase for each antibiotic.

### *In vitro* susceptibility testing

The *in vitro* antibacterial activity of β-lactams alone or in combination with β-lactamase inhibitors was determined in cation-adjusted Mueller Hinton broth (CAMHB) microdilution assays according to Clinical & Laboratory Standards Institute (CLSI) methods (77). Cefepime and ceftazidime were 2-fold serially diluted in CAMHB with or without a fixed concentration of 4 µg/mL of partner β-lactamase inhibitor, taniborbactam or avibactam, respectively (27). The inocula for the broth microdilution assays were prepared by the broth culture method in CAMHB supplemented with 10 µg/mL chloramphenicol to select for and maintain the chloramphenicol-resistant plasmid carrying the β-lactamase gene. The reported MICs are modal values from a minimum of three independent replicates. The quality control (QC) strains used were *E. coli* ATCC 25922 for every antibiotic tested in this study, *E. coli* NCTC 13353 for cefepime and cefepime-taniborbactam, and *K. pneumoniae* ATCC 700603 for ceftazidime, ceftazidime-avibactam, and aztreonam-avibactam, as described in the CLSI document M100-Ed31 (27) and the EUCAST document (78). MICs obtained for each antibiotic against the corresponding QC strains were within the QC ranges.

### *In silico* structure modeling and docking

The structure modeling and docking were performed using the Molecular Operating Environment software (MOE, Chemical Computing Group, version 2024.06). Homology models of IMP-4 and IMP-59 were constructed using reference sequences from the National Center for Biotechnology Information (NCBI) Identical Proteins database (IMP-4, WP_015060105.1; IMP-59, WP_094009805.1), and the X-ray structure coordinates for IMP-1 (PDB ID 5EV6) serving as the template. Non-covalent docking of taniborbactam was conducted using the derived homology models for IMP-4 and IMP-59, using a three-point pharmacophore model (B-OH as hydrogen-bond donor/acceptor, B-O-benzo ring as hydrogen-bond acceptor, benzo-ring as aromatic centroid) obtained from the VIM-2 co-crystal structure with taniborbactam (PDB 6SP7) to assist with positioning of the inhibitor. The highest-scoring docked poses for each were further minimized in the binding site, with the protein atoms constrained using the “tether” option.

## SUPPLEMENTAL MATERIAL

Supplemental material aac.01699-24-s0001.pdf Tables S1 to S4 and Figures S1 to S3.

ASM does not own the copyrights to Supplemental Material that may be linked to, or accessed through, an article. The authors have granted ASM a non-exclusive, world-wide license to publish the Supplemental Material files. Please contact the corresponding author directly for reuse.
