# Supplementary material for: Spectrum of cefepime-taniborbactam coverage against 190 β-lactamases defined in engineered isogenic Escherichia coli strains
Source: Antimicrob Agents Chemother. 2025 Apr 1;69(5):e01699-24. doi: 10.1128/aac.01699-24 (PMC12057355; doi:10.1128/aac.01699-24)
Supplement: Supplemental material — Tables S1 to S4 and Figures S1 to S3. [file aac.01699-24-s0001.pdf]

Supplemental Material

Spectrum of cefepime-taniborbactam coverage against 190 β-lactamases defined in engineered isogenic *Escherichia coli* strains

Tsuyoshi Uehara*, Cassandra L. Chatwin, Brittany Miller, Mitchel Edwards, Annie Stevenson, Jenna Colombo, David A. Six, Denis M. Daigle, Greg Moeck, Steven A. Boyd, and Daniel C. Pevear

Venatorx Pharmaceuticals, Inc., Malvern, PA, USA

Short running title: Cefepime-taniborbactam coverage against 190 β-lactamases

*Corresponding author: Tsuyoshi Uehara

Mailing Address: Venatorx Pharmaceuticals, Inc., 30 Spring Mill Drive, Malvern, PA 19355, USA

Phone: 484-329-8553

Email: uehara@venatorx.com

Table S1. Fold MIC increases against 190 β-lactamase-overproducing strains relative to vector control strains

E. coli DH10B producing	Ambler Class	Fold MIC increase relative to vector control							
		FEP	FEP-TAN	CAZ	CAZ-AVI	ATM	ATM-AVI	PIP	MEM
Signal peptide (pTU501 vector)	-	1	1	1	1	1	1	1	1
Signal peptide (pTU672 vector)*	-	1	1	1	1	1	1	1	1
TEM-10	A	32	8	>256	4	256	4	64	2
TEM-12	A	4	2	8	2	2	1	16	1
TEM-24	A	8	4	>256	16	64	4	4	2
TEM-72	A	128	2	>256	4	512	4	>64	8
CTX-M-2	A	512	2	64	4	512	2	>64	4
CTX-M-14*	A	128	1	8	1	128	1	>64	1
CTX-M-15	A	512	2	>256	2	>512	2	>64	4
CTX-M-219*	A	128	4	256	4	64	1	>64	2
GES-2*†	A	64	2	256	8	64	1	64	4
GES-4*†	A	4	1	128	4	16	2	64	16
GES-5†	A	8	2	128	8	8	1	>64	128
GES-6*†	A	32	2	>256	32	64	2	>64	128
GES-11*	A	512	2	>256	16	>512	4	>64	16
GES-13*	A	128	2	>256	32	>512	4	>64	8
GES-14*†	A	64	2	>256	8	32	1	>64	64
SHV-5	A	>512	2	>256	8	>512	8	>64	4
SHV-12	A	512	2	>256	8	>512	8	>64	4
VEB-9	A	512	2	>256	32	>512	16	32	1
VEB-14*	A	>1024	32	>256	>256	512	128	64	2
VEB-25*	A	>1024	4	>256	>256	>512	>512	>64	4
KPC-2†	A	256	2	128	2	>512	2	>64	512
KPC-21*†	A	32	2	128	2	>512	64	16	32
KPC-2 L168Q*†	A	64	4	256	8	64	4	32	16
KPC-2 del_172-175*†	A	2	2	32	16	2	1	2	1
KPC-14*†	A	256	16	>256	256	512	16	64	4
KPC-87*†	A	128	16	256	128	256	256	16	128
KPC-3†	A	512	2	>256	8	>512	2	>64	512
KPC-3 A177E†	A	512	2	>256	8	>512	2	>64	512

E. coli DH10B producing	Ambler Class	Fold MIC increase relative to vector control							
		FEP	FEP-TAN	CAZ	CAZ-AVI	ATM	ATM-AVI	PIP	MEM
KPC-31†	A	128	8	>256	>256	64	4	32	8
KPC-8†	A	512	4	>256	128	>512	8	>64	256
KPC-3 T243A†	A	256	2	>256	16	>512	2	>64	256
KPC-3 A177E D179Y†	A	64	4	>256	>256	32	8	32	8
KPC-32†	A	64	4	>256	256	32	2	32	8
KPC-66†	A	4	1	128	4	4	1	8	1
KPC-109*†	A	256	8	>256	256	256	4	>64	16
KPC-134*†	A	256	8	>256	>256	8	4	16	8
KPC-163*†	A	256	8	>256	256	256	4	64	8
PER-1	A	256	2	>256	32	>512	64	64	4
PER-2	A	>512	4	>256	128	>512	256	>64	4
PER-4*	A	512	16	>256	>256	>512	>512	8	1
PER-6*	A	1024	4	>256	128	>512	256	>64	4
PER-7*	A	1024	2	>256	64	>512	128	>64	4
PER-14*	A	64	2	>256	256	64	64	2	0.5
BEL-1*	A	4	1	128	1	128	1	>64	1
BEL-2*	A	32	1	128	4	128	1	16	1
IMI-1*†	A	32	2	32	4	>512	16	>64	1024
NmcA*†	A	64	2	16	4	>512	32	>64	2048
SFC-1*†	A	128	2	32	2	>512	4	>64	1024
SME-2*†	A	64	2	128	4	>512	32	>64	1024
LAP-2	A	1	2	2	1	2	1	8	1
NDM-1†	B	512	16	>256	>256	2	1	>64	1024
NDM-3†	B	>512	64	>256	>256	1	1	>64	>4096
NDM-5†	B	>512	64	>256	>256	2	1	>64	>4096
NDM-7†	B	>512	64	>256	>256	2	1	>64	>4096
NDM-9†	B	>512	>1024	>256	>256	1	1	>64	4096
NDM-10†	B	128	16	>256	>256	2	1	16	512
NDM-12†	B	>512	8	>256	>256	1	1	>64	>4096
NDM-13†	B	>512	64	>256	>256	1	1	>64	>4096
NDM-15†	B	>512	64	>256	>256	1	1	>64	>4096
NDM-16b†	B	>512	64	>256	>256	1	1	>64	>4096

E. coli DH10B producing	Ambler Class	Fold MIC increase relative to vector control							
		FEP	FEP-TAN	CAZ	CAZ-AVI	ATM	ATM-AVI	PIP	MEM
NDM-30*†	B	256	64	>256	>256	2	1	>64	1024
VIM-1†	B	>512	32	>256	>256	1	1	>64	1024
VIM-2†	B	16	1	256	256	1	1	>64	256
VIM-4†	B	128	2	>256	>256	1	1	>64	512
VIM-5†	B	16	2	>256	>256	1	1	>64	1024
VIM-7†	B	16	16	128	128	1	1	>64	1024
VIM-12†	B	>512	32	>256	>256	1	1	>64	1024
VIM-13†	B	32	2	>256	>256	2	1	>64	256
VIM-18†	B	128	64	>256	>256	1	1	>64	64
VIM-23†	B	256	8	>256	>256	1	1	>64	512
VIM-24†	B	256	8	>256	256	1	1	64	256
VIM-25†	B	16	2	>256	>256	1	1	>64	1024
VIM-26†	B	512	32	>256	>256	1	1	>64	1024
VIM-32†	B	256	16	>256	>256	2	1	>64	2048
VIM-83*†	B	>1024	>1024	>256	>256	1	1	>64	512
SPM-1†	B	256	16	>256	>256	2	1	>64	1024
GIM-1†	B	16	2	>256	>256	1	1	>64	512
SIM-1†	B	128	512	>256	>256	2	2	16	512
IMP-1†	B	256	512	>256	>256	1	1	16	512
IMP-4†	B	256	512	>256	>256	2	1	8	512
IMP-59*†	B	1024	128	>256	>256	2	2	32	1024
DIM-1*†	B	2	1	32	64	1	1	16	16
FIM-1*†	B	64	8	>256	>256	1	1	>64	512
TMB-1*†	B	4	1	32	32	1	0.5	8	16
ACT-1*	C	8	1	128	1	256	2	16	8
ACT-17	C	8	2	>256	2	256	4	64	2
ACT-86	C	512	32	>256	32	128	4	>64	≤2
ACT-C189 (P99/AmpC)	C	16	2	>256	2	256	4	>64	4
ACT-C189 del_293-294	C	512	64	>256	16	128	4	64	2
ACT-C191	C	512	64	>256	32	64	2	64	2
CMH-3	C	64	2	>256	256	256	4	>64	128
CMH-ENT385	C	256	8	>256	8	256	2	64	4

E. coli DH10B producing	Ambler Class	Fold MIC increase relative to vector control							
		FEP	FEP-TAN	CAZ	CAZ-AVI	ATM	ATM-AVI	PIP	MEM
CMH-ENT385-reversed	C	4	2	>256	2	256	4	32	4
CMY-2	C	8	1	>256	2	256	2	64	2
CMY-6	C	16	2	>256	2	512	4	64	8
CMY-16	C	16	1	>256	4	>512	4	>64	8
CMY-16 Y150S	C	4	2	32	8	>512	256	16	1
CMY-16 N346H	C	8	2	>256	64	>512	128	32	2
CMY-42	C	16	2	>256	4	>512	8	64	8
CMY-172*	C	1024	4	>256	256	256	8	64	16
CMY-185*	C	32	32	>256	>256	256	256	2	1
DHA-1*	C	1	1	64	1	16	1	16	1
FOX-4*	C	64	2	>256	128	512	16	64	16
FOX-5*	C	4	1	64	2	8	1	2	1
MIR-17*	C	4	2	128	1	256	4	16	8
MOX-1*	C	4	2	64	2	128	4	8	8
MOX-2*	C	16	2	>256	16	128	8	8	32
MOX-9*	C	32	1	256	1	64	2	8	2
PAC-1	C	512	256	>256	>256	128	64	32	8
PDC-1	C	8	2	128	2	128	2	>64	4
PDC-3	C	8	2	128	2	128	2	>64	2
PDC-5	C	8	2	128	2	128	2	>64	4
PDC-5 G183D	C	2	4	128	16	16	4	8	1
PDC-37	C	4	2	64	2	64	2	>64	4
PDC-50	C	16	2	>256	4	512	8	>64	4
PDC-73	C	4	1	128	4	64	1	>64	1
PDC-74	C	16	4	>256	4	>512	16	32	4
PDC-75	C	16	2	>256	4	>512	8	32	4
PDC-80	C	4	2	128	2	32	1	64	1
PDC-81	C	16	2	>256	4	256	2	>64	4
PDC-82	C	8	2	256	4	64	2	64	2
PDC-86	C	8	2	>256	8	256	2	32	2
PDC-87	C	8	2	>256	8	64	2	32	4
PDC-88	C	128	2	>256	2	64	1	>64	4

E. coli **DH10B producing**	**Ambler Class**	**Fold MIC increase relative to vector control**							
		**FEP**	**FEP-TAN**	**CAZ**	**CAZ-AVI**	**ATM**	**ATM-AVI**	**PIP**	**MEM**
PDC-89	C	128	4	64	4	64	2	16	4
PDC-90	C	256	4	>256	4	64	2	32	8
PDC-91	C	256	4	256	4	64	2	32	2
PDC-92	C	256	4	>256	4	64	2	64	4
PDC-221	C	2	2	128	4	16	2	4	1
PDC-222	C	4	4	128	8	16	4	8	1
PDC-223	C	4	4	256	16	16	2	8	1
PDC-237	C	32	2	>256	32	256	4	32	4
OXA-1	D (OXA-1)	128	128	2	1	4	1	>64	4
OXA-1 H109Q	D (OXA-1)	4	4	1	1	2	1	8	1
OXA-31*	D (OXA-1)	128	64	1	1	2	1	64	4
OXA-47*	D (OXA-1)	64	32	2	2	8	4	64	2
OXA-224*	D (OXA-1)	128	64	1	1	2	1	>64	2
OXA-2*	D (OXA-2)	8	1	128	2	2	1	4	32
OXA-3*	D (OXA-2)	2	2	8	1	2	1	>64	1
OXA-32*	D (OXA-2)	1	1	4	1	1	1	1	1
OXA-34*	D (OXA-2)	16	16	256	64	16	1	4	2
OXA-141*	D (OXA-2)	2	2	32	4	2	1	2	1
OXA-161*	D (OXA-2)	2	1	16	8	1	2	2	1
OXA-210*	D (OXA-2)	2	1	64	1	2	0.5	1	0.5
OXA-5*	D (OXA-5)	16	1	2	1	16	1	64	2
OXA-9*	D (OXA-9)	32	2	16	1	128	2	>64	4
OXA-10*	D (OXA-10)	64	1	4	2	128	16	>64	8
OXA-11*	D (OXA-10)	256	8	>256	>256	128	16	64	4
OXA-13*	D (OXA-10)	64	1	8	2	64	8	>64	2
OXA-14*	D (OXA-10)	128	4	>256	>256	256	16	>64	4
OXA-16*	D (OXA-10)	256	8	>256	>256	128	8	32	4
OXA-17*	D (OXA-10)	64	2	8	2	64	4	>64	4
OXA-19*	D (OXA-10)	32	1	>256	64	64	8	64	2
OXA-28*	D (OXA-10)	128	8	>256	256	128	16	32	4
OXA-35*	D (OXA-10)	64	1	4	2	128	16	>64	16
OXA-56*	D (OXA-10)	64	2	4	2	128	32	>64	16
OXA-74*	D (OXA-10)	128	2	16	4	256	32	>64	16

E. coli DH10B producing	Ambler Class	Fold MIC increase relative to vector control							
		FEP	FEP-TAN	CAZ	CAZ-AVI	ATM	ATM-AVI	PIP	MEM
OXA-142*	D (OXA-10)	128	4	>256	256	64	8	64	4
OXA-145*	D (OXA-10)	128	4	>256	>256	64	8	32	4
OXA-147*	D (OXA-10)	4	1	32	8	8	2	2	0.5
OXA-183*	D (OXA-10)	128	4	>256	>256	128	8	32	4
OXA-240*	D (OXA-10)	64	2	4	2	128	16	>64	16
OXA-256*	D (OXA-10)	64	2	16	4	64	8	64	8
OXA-18*	D (OXA-18)	1024	8	>256	4	>512	16	>64	16
OXA-23†	D (OXA-23)	32	32	1	1	2	1	>64	64
OXA-57*	D (OXA-42)	1	1	1	1	2	1	32	2
OXA-59	D (OXA-42)	1	2	1	1	2	2	>64	8
OXA-46*	D (OXA-46)	4	1	32	1	2	1	32	4
OXA-48†	D (OXA-48)	8	2	2	1	2	2	>64	128
OXA-162†	D (OXA-48)	16	2	4	1	2	1	>64	64
OXA-163	D (OXA-48)	256	2	>256	8	>512	4	>64	16
OXA-181†	D (OXA-48)	8	2	2	1	2	1	>64	64
OXA-204†	D (OXA-48)	4	1	2	1	1	1	>64	32
OXA-232†	D (OXA-48)	8	2	2	1	2	1	>64	64
OXA-244†	D (OXA-48)	4	2	4	1	4	1	>64	64
OXA-245†	D (OXA-48)	4	1	2	1	2	1	>64	64
OXA-247*†	D (OXA-48)	2	1	4	1	16	2	>64	2
OXA-370*	D (OXA-48)	2	2	1	0.5	2	0.5	32	8
OXA-405*	D (OXA-48)	64	1	128	2	64	2	>64	4
OXA-436*†	D (OXA-48)	2	1	1	1	1	1	>64	64
OXA-484†	D (OXA-48)	8	2	8	2	4	1	>64	64
OXA-517*†	D (OXA-48)	128	2	256	4	64	2	>64	64
OXA-519*†	D (OXA-48)	1	1	1	1	1	1	32	128
OXA-50	D (OXA-50)	1	2	1	1	2	1	2	4
OXA-396	D (OXA-50)	1	2	2	1	16	4	2	8
OXA-51†	D (OXA-51)	1	1	1	1	1	1	32	16
OXA-66†	D (OXA-51)	1	2	2	2	1	1	16	8
OXA-58†	D (OXA-58)	1	2	2	1	1	1	>64	16
OXA-427*†	D (OXA-427)	64	2	>256	16	256	8	>64	16

E. coli DH10B producing	Ambler Class	Fold MIC increase relative to vector control							
		FEP	FEP-TAN	CAZ	CAZ-AVI	ATM	ATM-AVI	PIP	MEM
LCR-1*	D	2	2	2	2	4	2	8	4

The fold increases of MICs shown in Table 1 were shown compared to the MIC against the corresponding vector control. β-lactamases were produced from either pTU501 or pTU672. β-lactamases produced from pTU672 are marked as an asterisk (*). β-lactamases reported as carbapenemase (KPCs, certain GES variants, SME, OXA-48 types, OXA-51 types, MBLs) at Beta-Lactamase DataBase and in literature are marked as a dagger (†). Taniborbactam and avibactam were tested in combination with cefepime and ceftazidime at a fixed concentration of 4 μg/mL each. In Ambler class D, OXA enzymes are listed as the groups of enzyme types. Abbreviations: ATM, aztreonam; AVI, avibactam; CAZ, ceftazidime; FEP, cefepime; MEM, meropenem; NA, not applicable; PIP, piperacillin; TAN, taniborbactam.

Table S2. Distribution of fold MIC increases against β-lactamase-overproducing strains relative to vector control strains

All classes of β-lactamases (n = 190)

Compound	MIC fold increase from the vector control									
	**1***	**2**	**4**	**8**	**16**	**32**	**64**	**128**	**256**	**>256**
FEP	11	12	21	17	17	12	21	23	21	35
Cumulative %	5.8	12.1	23.2	32.1	41.1	47.4	58.4	70.5	81.6	100
FEP-TAN	34	82	23	14	9	8	12	2	1	5
Cumulative %	17.9	61.1	73.2	80.5	85.3	89.5	95.8	96.8	97.4	100
CAZ	11	13	8	6	5	9	7	21	11	99
Cumulative %	5.8	12.6	16.8	20.0	22.6	27.4	31.1	42.1	47.9	100
CAZ-AVI	35‡	30	28	16	9	8	5	6	11	42
Cumulative %	18.4	34.2	48.9	57.4	62.1	66.3	68.9	72.1	77.9	100
ATM	31	32	5	5	10	4	26	20	20	37
Cumulative %	16.3	33.2	35.8	38.4	43.7	45.8	59.5	70.0	80.5	100
ATM-AVI	76	39	28	17	12	4	4	3	5	2
Cumulative %	40.0	60.5	75.3	84.2	90.5	92.6	94.7	96.3	98.9	100

*The number ‘1’ means the same MIC against the engineered strain and the vector control.

‡There was one instance for a class D-producing strain (OXA-370) with one doubling dilution below the vector.

Abbreviations: ATM, aztreonam; AVI, avibactam; CAZ, ceftazidime, FEP, cefepime; TAN, taniborbactam.

Table S3. Distribution of fold MIC increases against β-lactamase-overproducing strains relative to vector control strains, categorized by ambler class

Class A β-lactamases (n = 50)

Compound	MIC fold increase from the vector control									
	**1***	**2**	**4**	**8**	**16**	**32**	**64**	**128**	**256**	**>256**
FEP	1	1	4	2		5	8	7	7	15
Cumulative %	2	4	12	16		26	42	56	70	100
FEP-TAN	5	27	9	5	3	1				
Cumulative %	10	64	82	92	98	100				
CAZ		1		2	1	3	1	8	4	30
Cumulative %		2		6	8	14	16	32	40	100
CAZ-AVI	3	5	10	8	4	4	1	4	5	6
Cumulative %	6	16	36	52	60	68	70	78	88	100
ATM		3	1	2	1	3	7	3	4	26
Cumulative %		6	8	12	14	20	34	40	48	100
ATM-AVI	11	9	11	4	3	2	3	2	3	2
Cumulative %	22	40	62	70	76	80	86	90	96	100

Class B β-lactamases (n = 34)

Compound	MIC fold increase from the vector control									
	**1***	**2**	**4**	**8**	**16**	**32**	**64**	**128**	**256**	**>256**
FEP		1	1		5	1	1	4	7	14
Cumulative %		2.9	5.9		20.6	23.5	26.5	38.2	58.8	100
FEP-TAN	3	5		4	5	3	8	1		5
Cumulative %	8.8	23.5		35.3	50	58.8	82.4	85.3		100
CAZ						2		1	1	30
Cumulative %						5.9		8.8	11.8	100
CAZ-AVI						1	1	1	2	29
Cumulative %						2.9	5.9	8.8	14.7	100
ATM	23	11								
Cumulative %	67.6	100								
ATM-AVI	32	2								
Cumulative %	94.1	100								

Class C β-lactamases (n = 48)

Compound	MIC fold increase from the vector control									
	**1***	**2**	**4**	**8**	**16**	**32**	**64**	**128**	**256**	**>256**
FEP	1	2	10	10	9	3	2	2	4	5
Cumulative %	2.1	6.3	27.1	47.9	66.7	72.9	77.1	81.3	89.6	100
FEP-TAN	7	26	9	1		2	2		1	
Cumulative %	14.6	68.8	87.5	89.6		93.8	97.9		100	
CAZ						1	5	10	4	28
Cumulative %						2.1	12.5	33.3	41.7	100
CAZ-AVI	4	13	13	5	4	3	1	1	2	2
Cumulative %	8.3	35.4	62.5	72.9	81.3	87.5	89.6	91.7	95.8	100
ATM				1	5	1	11	8	13	9
Cumulative %				2.1	12.5	14.6	37.5	54.2	81.3	100
ATM-AVI	5	19	13	5	2		1	1	2	
Cumulative %	10.4	50	77.1	87.5	91.7		93.8	95.8	100	

Class D β-lactamases (n = 58)

Compound	MIC fold increase from the vector control									
	**1***	**2**	**4**	**8**	**16**	**32**	**64**	**128**	**256**	**>256**
FEP	9	8	6	5	3	3	10	10	3	1
Cumulative %	15.5	29.3	39.7	48.3	53.4	58.6	75.9	93.1	98.3	100
FEP-TAN	19	24	5	4	1	2	2	1		
Cumulative %	32.8	74.1	82.8	89.7	91.4	94.8	98.3	100		
CAZ	11	12	8	4	4	3	1	2	2	11
Cumulative %	19	39.7	53.4	60.3	67.2	72.4	74.1	77.6	81	100
CAZ-AVI	28[‡]	12	5	3	1		2		2	5
Cumulative %	48.3	69	77.6	82.8	84.5		87.9		91.4	100
ATM	8	18	4	2	4		8	9	3	2
Cumulative %	13.8	44.8	51.7	55.2	62.1		75.9	91.4	96.6	100
ATM-AVI	28	9	4	8	7	2				
Cumulative %	48.3	63.8	70.7	84.5	96.6	100				

*'1' means the same MIC against the engineered strain and the vector control.

[‡]There was one instance for a strain (OXA-370) with one doubling dilution below the vector. Abbreviations: ATM, aztreonam; AVI, avibactam; CAZ, ceftazidime, FEP, cefepime; TAN, taniborbactam.

Table S4. Effect of the IMP N185Y substitution on taniborbactam activity

Plasmid	*E. coli* DH10B producing	Cefepime							Ceftazidime		ATM	PIP	MEM
		-	TAN [4]	TAN [8]	LED [4]	LED [8]	AVI [4]	VAB [8]	-	AVI [4]			
**pTU501**	**Signal peptide**	0.25	0.12	0.12	0.12	0.12	0.12	0.12	0.5	0.5	0.5	4	≤0.06
**pTU672**	**Signal peptide**	0.12	0.12	0.12	0.12	0.12	0.12	0.12	0.5	0.5	0.25	2	≤0.06
**pTU538**	**IMP-4**	64	32	32	32	32	64	32	>128	>128	0.25	16	16
**pTU796***	**IMP-59 (IMP-4_N233Y)**	128	16	4	32	16	128	128	>128	>128	0.5	64	16
**pTU535**	**IMP-1**	64	32	32	64	32	64	64	>128	>128	0.5	32	16
**pTU797***	**IMP-1_N233Y**	64	8	4	16	8	64	64	>128	>128	0.5	64	32

All values are in μg/mL.

*β-lactamases produced from pTU672 are marked as an asterisk.

β-lactamase inhibitors, taniborbactam (TAN), ledaborbactam (LED), avibactam (AVI), and vaborbactam (VAB), were tested in combination with cefepime and ceftazidime at a fixed concentration of 4 or 8 μg/mL.

Abbreviations: ATM, aztreonam; AVI, avibactam; CAZ, ceftazidime; FEP, cefepime; MEM, meropenem; NA, not applicable; PIP, piperacillin; TAN, taniborbactam.

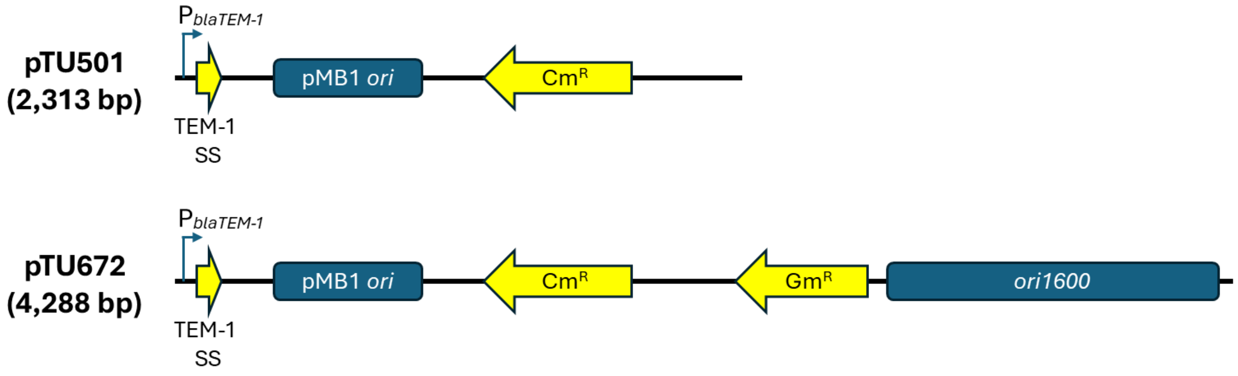

Figure S1. Maps of the vector plasmids pTU501 and pTU672.

A DNA fragment encoding the mature periplasmic moiety of each β-lactamase was cloned just downstream of the coding frame of the TEM-1 signal sequence (TEM-1 SS) for expression in the periplasm using the signal sequence. Both pTU501 and pTU672 share the TEM-1 promoter ($P_{\mathit{bla}\text{TEM-1}}$), TEM-1 SS, a pMB1 origin supporting replication in *E. coli*, and the chloramphenicol resistant gene (Cm^R). Compared to pTU501, pTU672 carries an origin of replication in *P. aeruginosa* (*ori1600*) and a gentamicin resistant gene (Gm^R).

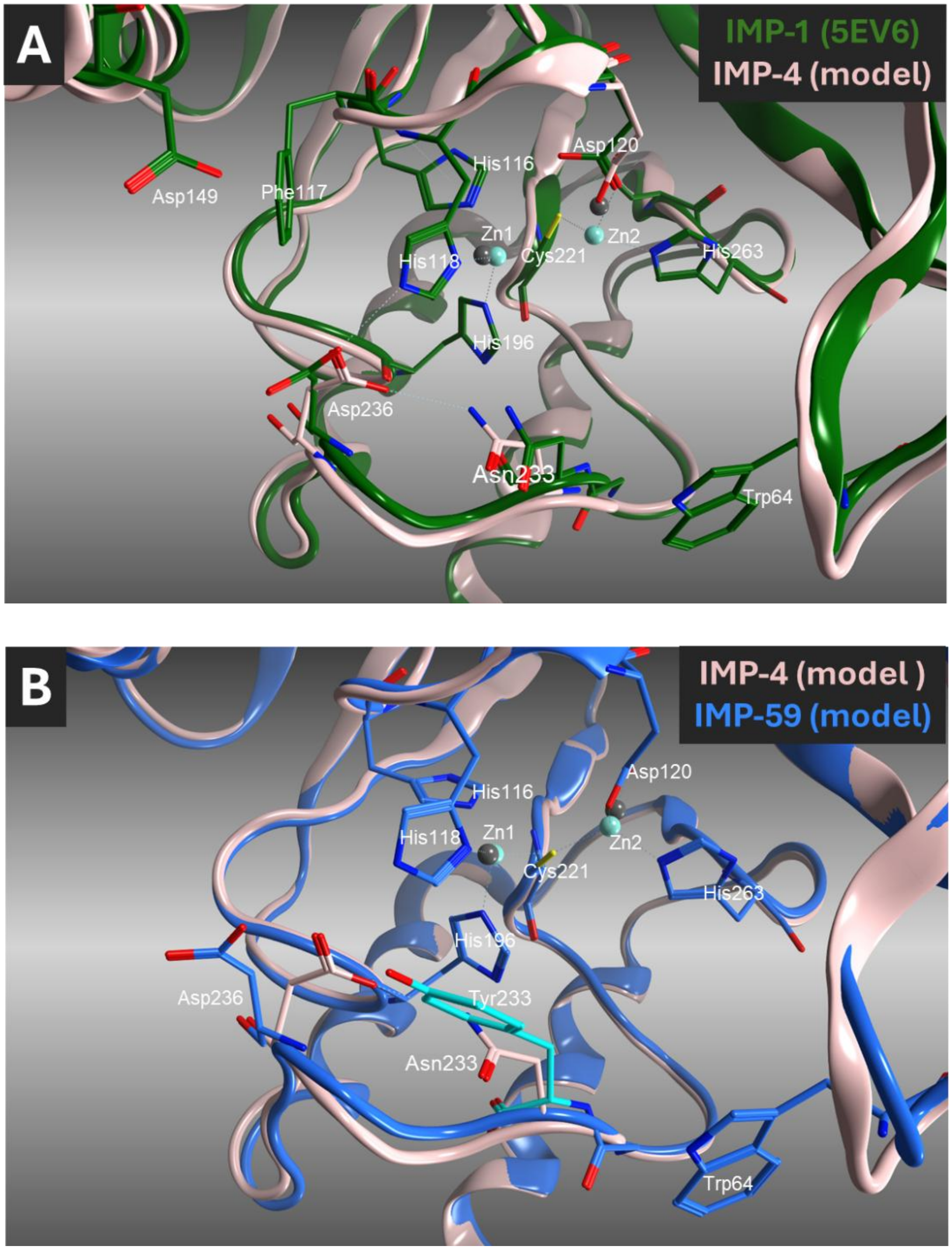

Figure S2. Homology models of IMP-4 and IMP-59.

A. The homology model of IMP-4 [NCBI RefSeq WP_015060105.1] was built using the Molecular Operating Environment software package (MOE, Chemical Computing Group, version 2024.06), employing an IMP-1 crystal structure (PDB 5EV6) as the template (95% identity). The structures of IMP-1 (dark green) and IMP-4 (light pink) are superposed, showing 100% identity of residues within the active sites of these enzymes. Minor differences in the side chain conformations of

Asp120, Asn233, and Asp236 were obtained in the minimization process, but the binding sites are very similar. **B.** The homology model of IMP-59 was also built based on the IMP-1 crystal structure (PDB 5EV6), using the published sequence of IMP-59 [NCBI RefSeq WP_094009805.1] (94.6% identity). The single amino acid change within the binding site is Asn233 to Tyr (cyan), which affects the ligand binding region significantly by creating a more lipophilic region in place of the polar Asn side chain. Asp236 is rotated away from the binding region due to steric interactions with Tyr233. The residue numbers shown in **Figures S2** and **S3** follow the standard numbering scheme for MBLs (Galleni et al. 2001. *Antimicrob Agents Chemother*. 45:660-3. DOI: 10.1128/AAC.45.3.660-663.2001).

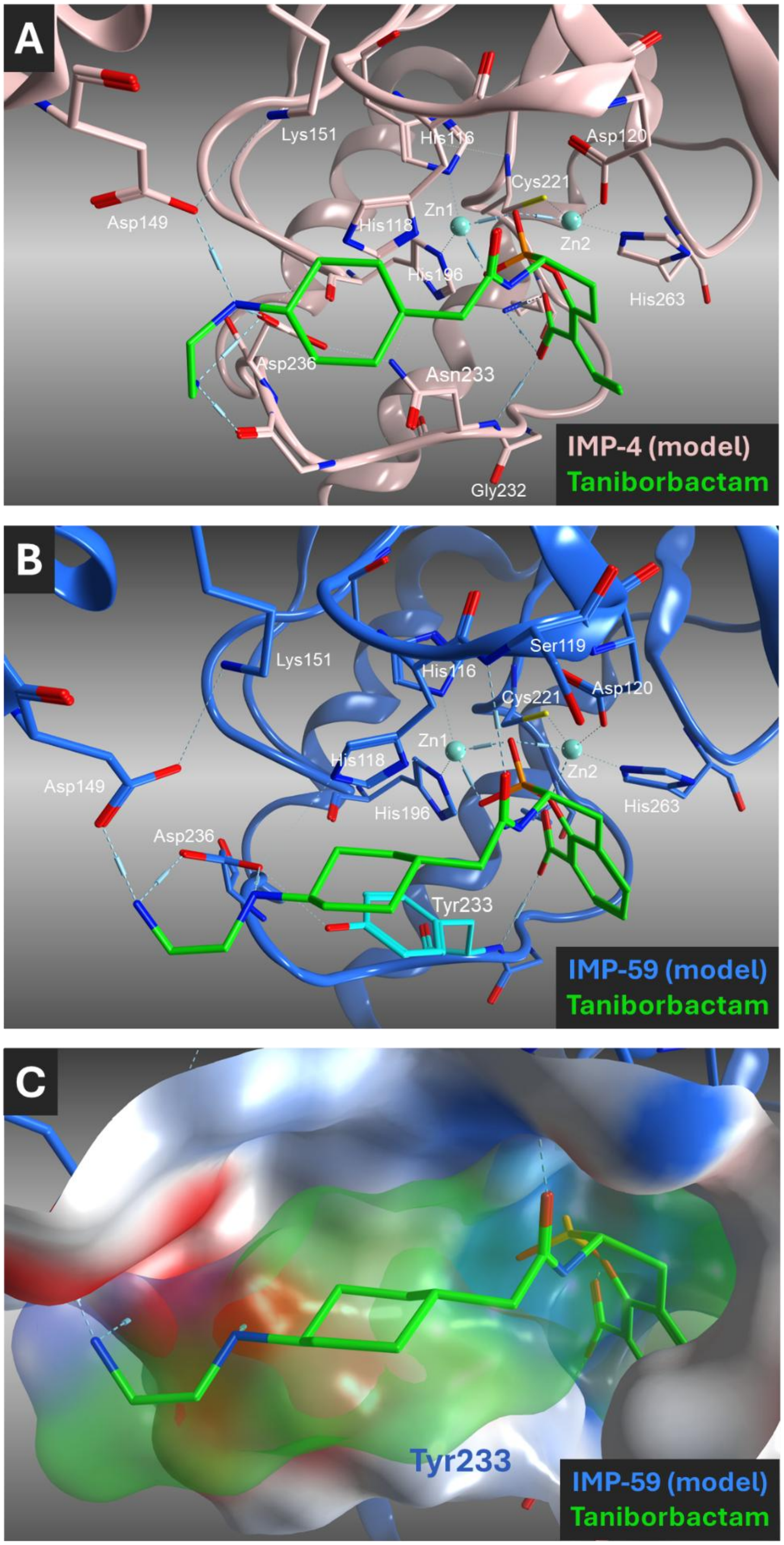
A
Lys151
His116
Asp120
Cys221
Asp149
Zn1
His118
Zn2
His196
His263
Asp236
Asn233
IMP-4 (model)
Taniborbactam
Gly232
B
Ser119
Lys151
His116
Asp120
Cys221
Zn1
Asp149
His118
Zn2
His196
His263
Asp236
Tyr233
IMP-59 (model)
Taniborbactam
C
Tyr233
IMP-59 (model)
Taniborbactam

Figure S3. Homology models of IMP-4 and IMP-59 in complex with taniborbactam.

The docking of taniborbactam into the model structures were performed using non-covalent docking in MOE, employing a three-point pharmacophore (B-OH as hydrogen-bond donor/acceptor, B-O-benzo ring as hydrogen-bond acceptor, benzo-ring as aromatic centroid) obtained from the VIM-2 co-crystal structure with taniborbactam (PDB 6SP7) to assist with positioning of the inhibitor. The highest-scoring docked poses were further minimized in the binding site, with the protein atoms constrained using the "tether" option. **A.** IMP-4 modeled structure in complex with taniborbactam, showing the cyclohexyl ring pushed out of the binding pocket by a hydrogen-bonding network between Asn233 and Asp236. **B.** IMP-59 modeled structure in complex with taniborbactam, showing the cyclohexyl-acetyl group of taniborbactam making extensive hydrophobic contacts with Tyr233. Residue numbers for the IMP-59 protein sequence are shown. **C**. Solvent-accessible surface rending of the complex, using Poisson-Boltzmann electrostatic coloring (blue = positively-charged surface, white = neutral surface, red = negatively charged surface). The surface for taniborbactam is shown as partly transparent green rendering, highlighting the close contacts with Tyr233. The Tyr233 residue in the standard numbering scheme for MBLs is equivalent to Tyr185 of the actual IMP-59 protein sequence.
